# Highly sensitive and adaptable fluorescence-quenched pair discloses the substrate specificity profiles in diverse protease families

**DOI:** 10.1038/srep43135

**Published:** 2017-02-23

**Authors:** Marcin Poreba, Aleksandra Szalek, Wioletta Rut, Paulina Kasperkiewicz, Izabela Rutkowska-Wlodarczyk, Scott J. Snipas, Yoshifumi Itoh, Dusan Turk, Boris Turk, Christopher M. Overall, Leszek Kaczmarek, Guy S. Salvesen, Marcin Drag

**Affiliations:** 1 Department of Bioorganic Chemistry, Faculty of Chemistry, Wroclaw University of Technology, Wyb. Wyspianskiego 27, 50-370 Wroclaw, Poland; 2 NCI-designated Cancer Center, Sanford-Burnham Prebys Medical Discovery Institute, La Jolla, CA 92037, USA; 3 Laboratory of Neurobiology, Nencki Institute of Experimental Biology, Polish Academy of Sciences, 02-093 Warsaw, Poland; 4 Kennedy Institute of Rheumatology, University of Oxford, United Kingdom; 5 Department of Biochemistry and Molecular and Structural Biology, Jožef Stefan Institute, SI-1000 Ljubljana, Slovenia; 6 Centre for Blood Research, Department of Oral Biological and Medical Sciences, University of British Columbia, Vancouver, BC V6T 1Z3, Canada

## Abstract

Internally quenched fluorescent (IQF) peptide substrates originating from FRET (Förster Resonance Energy Transfer) are powerful tool for examining the activity and specificity of proteases, and a variety of donor/acceptor pairs are extensively used to design individual substrates and combinatorial libraries. We developed a highly sensitive and adaptable donor/acceptor pair that can be used to investigate the substrate specificity of cysteine proteases, serine proteases and metalloproteinases. This novel pair comprises 7-amino-4-carbamoylmethylcoumarin (ACC) as the fluorophore and 2,4-dinitrophenyl-lysine (Lys(DNP)) as the quencher. Using caspase-3, caspase-7, caspase-8, neutrophil elastase, legumain, and two matrix metalloproteinases (MMP2 and MMP9), we demonstrated that substrates containing ACC/Lys(DNP) exhibit 7 to 10 times higher sensitivity than conventional 7-methoxy-coumarin-4-yl acetic acid (MCA)/Lys(DNP) substrates; thus, substantially lower amounts of substrate and enzyme can be used for each assay. We therefore propose that the ACC/Lys(DNP) pair can be considered a novel and sensitive scaffold for designing substrates for any group of endopeptidases. We further demonstrate that IQF substrates containing unnatural amino acids can be used to investigate protease activities/specificities for peptides containing post-translationally modified amino acids. Finally, we used IQF substrates to re-investigate the P1-Asp characteristic of caspases, thus demonstrating that some human caspases can also hydrolyze substrates after glutamic acid.

The irreversible peptide bond hydrolysis of proteins and polypeptides is the most conserved post-translational modification occurring in biochemical pathways in all living organisms^1^,^2^. This reaction is catalyzed by proteases, which specifically recognize protein targets to control numerous significant biological processes, including cell survival and cell death and the immune response to various pathogens^3^. The selectivity of proteases for binding and subsequently hydrolyzing a selected group of peptides or proteins is termed substrate specificity^4^,^5^. The increasing number of chemical tools for substrate specificity profiling allows the development of new, more efficient and more selective small molecule substrates^6^,^7^, inhibitors^8^, and chemical probes^9^, which are useful for the determination of protease activity and the dissection of their physiological functions.

Internally quenched fluorescent (IQF) peptide substrates constitute a convenient tool for examining the specificity of the largest group of proteases – endopeptidases^10^. These substrates contain a paired fluorophore (donor) and quencher (acceptor), which are located on opposite sides of the scissile peptide bond^11^,^12^. If the fluorophore emission spectrum and the quencher absorption spectrum overlap, intramolecular energy transfer occurs and quenches the fluorescence. Upon hydrolysis of the peptide bond, the distance between the donor and the acceptor increases, the fluorophore is no longer quenched, and an enhanced fluorescence signal is generated. IQF substrates are often called FRET (Förster Resonance Energy Transfer) substrates, however it must be stressed here that IQF is a special (more narrow) type of FRET molecule. In traditional internally quenched fluoresce substrates the acceptor (or quencher) is non-fluorescent, where in FRET substrates the acceptor can be either fluorescent or not^13^. The most commonly used IQF/FRET substrate pairs include Edans-Dabcyl^11^, ABz-Tyr(NO_2_)^14^, ABz-EDDNP^15^, Trp-Dansyl^16^, and 7-methoxy-coumarin-4-yl acetic acid-2,4-dinitrophenyl-lysine (MCA-Lys(DNP))^17^,^18^ (for more examples, please see review^19^). Although IQF substrates provide an opportunity to sample substrate preferences on both sides of the peptide bond, they tend to exhibit higher background fluorescence than simple peptidyl fluorophores (especially when ABz or MCA are used as fluorophores in IQF substrates). Therefore, there is a need to enhance the sensitivity by finding a donor/acceptor pair that yields a strong fluorescence signal and possesses good solubility, especially when using hydrophobic amino acids in peptide sequences. The 7-amino-4-carbamoylmethylcoumarin (ACC) fluorophore is widely used in simple fluorescent substrates in cases where non-prime site substrate specificity is examined and where ACC occupies the P1′ position^19^. However, only one detailed study has examined internally quenched substrates using ACC as a fluorophore (quenched with Dabcyl)^20^. This pair provides promising fluorescence, but the hydrophobic nature of the Dabcyl quencher poses solubility problems, and the high cost of Dabcyl significantly limits its application to the design of large substrate libraries. Another disadvantage of Dabcyl is its bulky structure, which can result in unexpected interactions with protease binding pockets. In contrast, the 2,4-nitrophenyl (DNP) moiety is inexpensive, is significantly smaller in size than Dabcyl, and is also a commonly used quencher that can easily be incorporated into peptide substrates; for example, in MCA substrates, DNP is often used to quench MCA. We speculated that the total fluorescence yield of ACC would be higher than that of MCA. In addition, ACC is slightly less hydrophobic than MCA, thus providing better solubility in aqueous environments (Fig. 1). Considering the aforementioned properties, we reasoned that an IQF pair comprising ACC and DNP would be optimal for a variety of endopeptidase assay applications.

To test our hypothesis, we designed several ACC/DNP IQF substrates based on known general sequence preferences utilizing the cysteine protease legumain, an inhibitor of osteoclast formation and bone resorption^21^, which is also overexpressed in many human solid tumors^22^ and caspases, which play main roles in the initiation and execution of apoptosis^23^. We also investigated the serine proteases trypsin and neutrophil elastase, the latter is involved in the first line of defense against pathogens and participates in the regulation of inflammatory processes through the processing of cytokines, chemokines, and growth factors and the activation of specific cell surface receptors^24^. Finally, we deployed IQF for matrix metalloproteinases (MMPs, specifically MMP2 MMP9), enzymes that are implicated in extracellular homeostasis, innate immunity, tumor cell migration and metastasis^25^,^26^,^27^ to explore the breadth and utility of the substrates. Having optimized the synthesis and characteristics of the novel IQF substrates, we used the obtained knowledge to address two topical questions in protease biology. First, we evaluated the specificity of caspases for Asp versus Glu at P1. This question regarding specificity arises because most studies assume that caspases are essentially totally selective for cleaving after Asp; however, several studies have found that some sequences are cleaved following Glu, thus potentially re-defining the target specificity of these physiologically important enzymes^28^.

The second question we asked was whether post-translationally modified residues can be effectively cleaved by trypsin. In a proof-of-concept experiment, we used trypsin, a protease that is commonly used in many proteomic workflows and other biochemical assays^29^,^30^,^31^. A previous study demonstrated that internally quenched substrates are suitable for trypsin assays^32^. For our purposes, we synthesized two trypsin-tailored (P1 = Arg or Lys) substrates and five analogues containing derivatized arginine or lysine at the same P1 position. This second question relates to the generation of proteomic profiles and whether modified Arg or Lys residues have been missed due to the focused tryptic searches for unmodified Arg and Lys residues in the vast majority of proteomic databases.

Finally, we used an 80-member internally quenched fluorogenic substrate library to revisit the P1 and P1′ subsite preferences of caspase, thus determining how stringent these proteases are for Asp versus Glu at P1 and whether the stringency for Asp or Glu is influenced by the nature of the P1′ residue.

## Results

### Spectroscopic analysis and quenching efficiency

**Figure 2:**
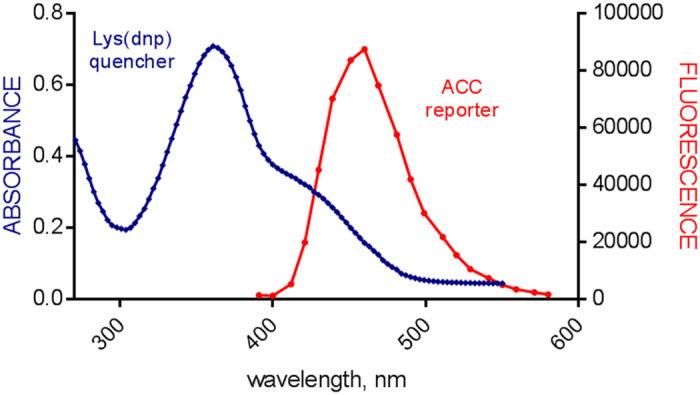
Spectral overlap of the DNP quenching group and the ACC fluorophore. The absorption spectrum of Lys(DNP) (blue) and the fluorescence emission spectrum of ACC (red).

**Figure 3:**
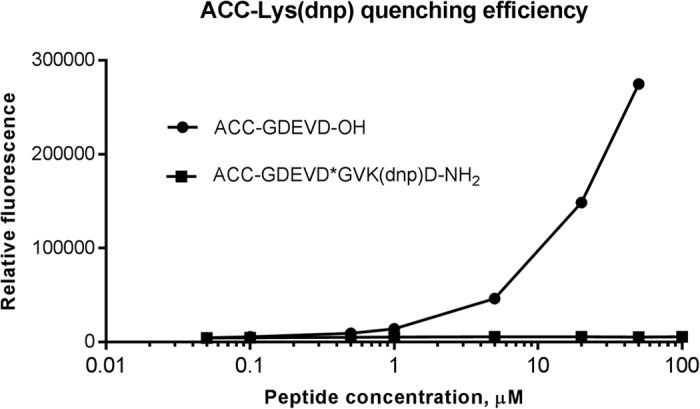
ACC fluorescence is efficiently quenched by the DNP moiety in the caspase substrate ACC-GDEVD*GVK(DNP)D. Before caspase-mediated hydrolysis, the ACC/DNP substrate emits almost no measurable fluorescence.

The key principle in designing IQF substrates is that the absorbance spectrum of the acceptor (quencher) must overlap with the fluorescence emission spectrum of the donor. We determined the absorption spectrum of the DNP group and the emission spectrum of the ACC fluorophore in four different assay buffers. Because the shapes of these spectra were similar, we show the results obtained in elastase buffer as an example (Fig. 2). Lys(DNP) has an absorbance maximum at 360 nm and an apparent shoulder to 500 nm. The ACC fluorescence emission presents a maximum at 460 nm, and the prominent shoulder in the absorption spectrum of DNP overlaps with the emission spectrum of ACC. To determine whether this spectral overlap is sufficient for ACC fluorophore quenching, we synthesized ACC-GDEVD-OH, a caspase substrate cleavage product, and compared its fluorescence with that of the full-length substrate ACC-GDEVD*GVK(DNP)D at various concentrations (Fig. 3). We observed almost no measurable fluorescence from ACC-GDEVD*GVK(DNP)D even at a very high concentration (100 μM). However, the fluorescence signal emitted by the ACC-GDEVD-OH cleavage product at a concentration of 100 μM exceeded the spectrofluorimeter scale. To further demonstrate that ACC/dnp quenching is efficient in our FRET substrates we measured the Förster distance (also called critical distance, R_0_) for this pair. The R_0_ value for the ACC/dnp pair was only slightly lower than MCA/dnp; 34.7 Å and 36.5 Å, respectively. Moreover, the fluorescence quantum yields (ϕF) for quenched substrates were negligible (0.00288 for ACC substrate and 0.00504 for MCA substrate) comparing to ϕF values for free fluorophores (0.861 for ACC and 0.718 for MCA).

### Internally quenched substrate architecture

**Figure 4:**
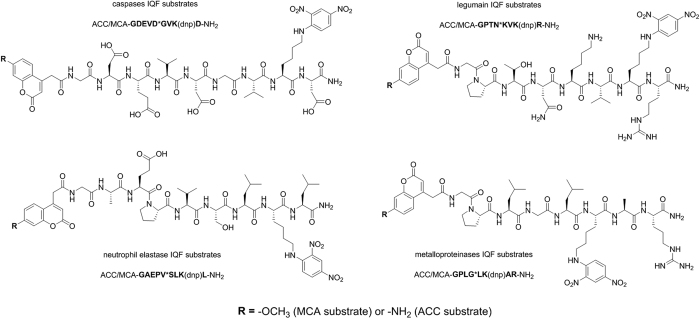
Structures of internally quenched substrates containing the classic MCA/DNP pair and the new ACC/DNP donor/acceptor pair.

To determine the usefulness of the new ACC/DNP pair and to compare it with the classic MCA/DNP pair, we synthesized matched substrates and analyzed them with seven proteolytic enzymes representing three mechanistically and catalytically different groups (cysteine proteases, serine proteases and metalloproteases). The substrates contained eight- or nine-amino acid main chains that were N-terminally labeled with either ACC or MCA and were quenched with Lys(DNP). This quencher was placed at the P2′ or P3′ position to ensure efficient energy transfer from the fluorophore molecules because the distance separating the donor and the acceptor is usually 10–100 Å^33^. To obtain high proteolytic activity in the kinetic assay, we selected substrates with optimal sequences for each enzyme: for caspases, we chose the optimal DEVD*G^34^ motif by synthesizing fluorophore-GDEVD*GVK(DNP)D substrates; for legumain, we synthesized fluorophore-GPTN*KVK(DNP)R substrates that contained a P3-P1 PTN sequence that was determined to be optimal for legumain^35^ -for human neutrophil elastase, the substrate contained the preferred AEPV motif in the P4-P1 region^9^ for legumain and elastase, the amino acids in the prime region of the substrates were chosen based on an analysis of substrate specificity profiles that have been deposited in the MEROPS database^5^ and finally, for MMP2, the substrate sequence (fluorophore-GPLG*LK(DNP)AR) was designed based on a previously reported MCA/Dpa-labeled substrate^36^. Due to the high degree of overlap in the substrate specificities of MMP2 and MMP9, the same substrates were used for these two enzymes^37^. In each substrate, Gly was included as a short, neutral linker between the fluorophore and the peptide recognition sequence. The structures of these substrates are presented in Fig. 4.

### IQF substrate sensitivity assay

**Figure 5:**
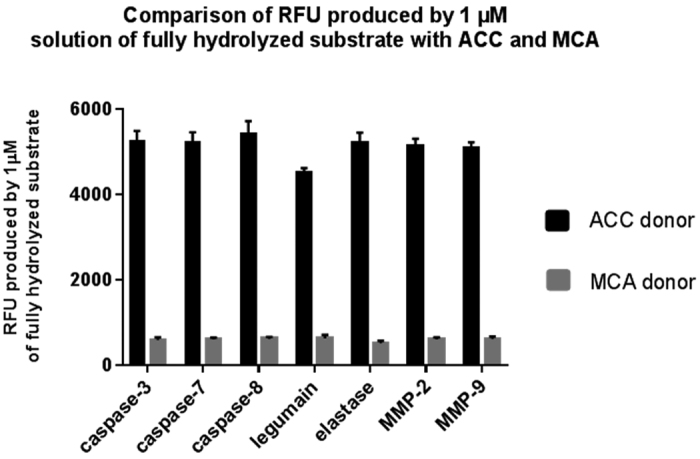
Comparison of the relative fluorescent units (RFUs) produced by 1 μM solutions of the hydrolyzed substrates containing ACC (black bars) or MCA (grey bars) in various assay buffers and for various substrate-protease pairs.

To determine whether our new donor/acceptor pair provides a strong signal, we measured the fluorescence produced by 1 μM solutions of the hydrolyzed substrates containing the new donor/acceptor pair ACC/DNP and compared it with the fluorescence obtained for the commonly used MCA/DNP pair at the optimal excitation and emission wavelengths for each donor/acceptor pair (ACC donor: 355 nm excitation and 460 nm emission; MCA donor: 325 nm excitation and 420 nm emission). These wavelengths were selected by screening the entire excitation and emission spectra for both fluorophores. The use of ACC/DNP improved the assay sensitivity approximately 10-fold (Fig. 5). The fluorescence increased by 7.2-fold to 10.1-fold for the tested enzymes and depended on the buffer used in the assay.

### Kinetic parameters

We next measured the kinetic parameters (K_m_, k_cat_, and k_cat_/K_m_) of the substrates with various proteases (Table 1). For all substrates, the new donor acceptor pair ACC/DNP had k_cat_/K_M_ values that were within a factor of two of those for the substrates containing MCA/DNP; caspase-3 and caspase-7 favored MCA/DNP, and elastase and MMP2 favored ACC/DNP. We observed no substantial differences for the individual values of K_m_ and k_cat_ between the matched substrates; thus, we conclude that positioning either fluorophore (ACC or MCA) at the N-terminal end of the substrates (at the P5 or P6 position) outside the protease active site does not differentially affect enzyme kinetics.

### Dissection of post-translational modifications using synthetic substrates – assays using trypsin

**Figure 6:**
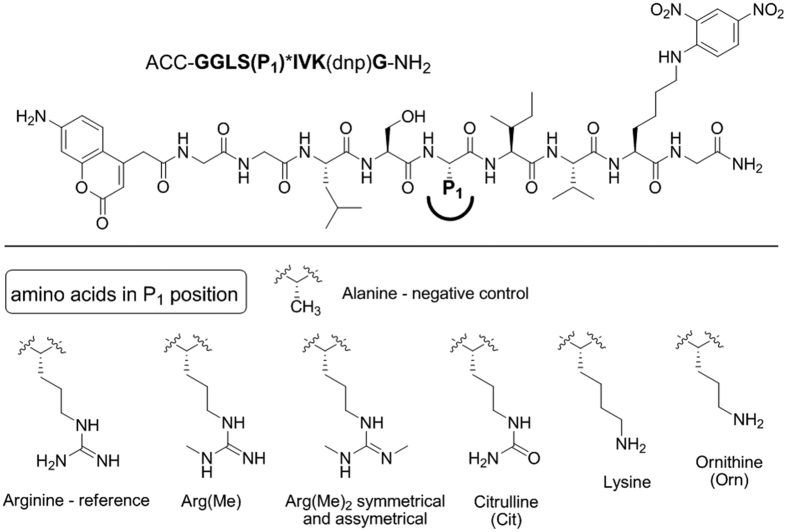
Structures of the eight internally quenched substrates that were used to evaluate the preferences of trypsin for post-translationally modified arginine and lysine residues at the P1 position.

Trypsin is the most widely used enzyme for digesting proteins into short peptides in mass spectrometry proteomics-based research. This enzyme is catalytically very active and is thought to cleave its substrates exclusively after positively charged lysine and arginine (P1 position) residues. Moreover, trypsin prefers substrates with cleavage sites that are surrounded by non-polar amino acids and produces peptides that are easily ionizable (due to the presence of protonated Arg and Lys residues) and that are in the preferred mass range (10–12 residues) for effective fragmentation in tandem mass spectrometry^30^,^31^. These properties strongly facilitate extensive proteome digestion and subsequent bioinformatics analysis^29^. Despite the strong preferences of trypsin for cleaving substrates after lysine and arginine, it has recently been reported that tryptic digestion can also occur after a ubiquitinated lysine^38^. This observation strongly supports the hypothesis that tryptic digestion can also generate protein fragments that do not contain Lys/Arg at their C-termini. Utilizing the novel IQF substrates, we examined whether trypsin can cleave substrates after post-translationally modified lysine and arginine residues. This question is particularly pertinent for the proteomic analyses of epigenetic protein markers in which both residues are modified. We synthesized eight internally quenched substrates; we selected the cleavage site of chymotrypsinogen A as a peptide scaffold and synthesized substrates with the general formula ACC-GGLSX*IVK(DNP)G, where X is methylated arginine, citrulline or ornithine in the P1 position. We employed a substrate containing Ala at P1 as a negative control. The panel of trypsin-targeted substrates is presented in Fig. 6; kinetic analysis revealed that trypsin almost exclusively preferred unmodified Arg and Lys at P1 (k_cat_/K_m_ values – 9,624,000 M^−1^s^−1^ and 1,500,000 M^−1^s^−1^) and did not recognize Ala. We observed extremely slow hydrolysis for singly and doubly methylated arginines, as recently reported^39^, and for citrulline and ornithine (Table 2); the obtained k_cat_/K_m_ values revealed that monomethyl Arg-containing substrates are cleaved at approximately 2 × 10^−4^ the rate obtained for their unmodified Arg-containing counterparts, and the activity for all other derivatives was 5 × 10^6^ times lower than for the unmodified substrates. Importantly, hydrolysis rates (comparing substrates containing Arg/Lys and their derivatives) were mainly determined by k_cat_, consistent with the binding of the substrate to the cleft but with a non-productive alignment of the scissile bond. We conclude that although trypsin can hydrolyze substrates after modified monomethylated arginine at a slow rate, this hydrolysis may result in low numbers of peptides that may have been previously overlooked in proteomic analyses. However, other proteases, such as LysargiNase, are highly efficient at hydrolyzing such substrates^39^.

### Caspase P1/P1′ specificity revisited

**Figure 7:**
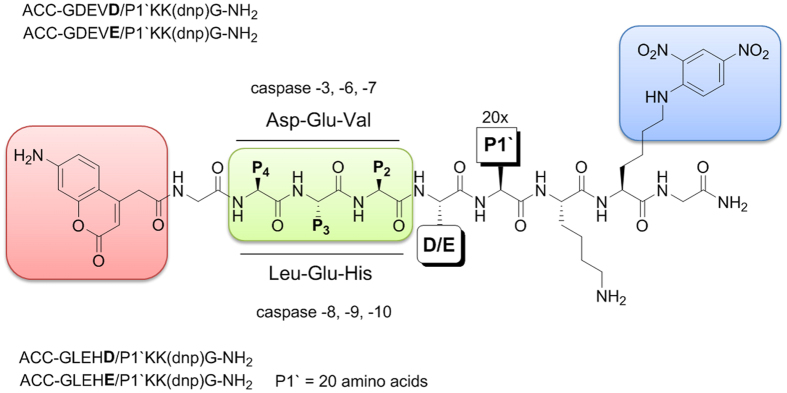
Overall architecture of the internally quenched substrates used to dissect caspase P1-P1′ specificity requirements.

As their name indicates, caspases are cysteine proteases that cleave substrates after aspartic acid residues^23^,^40^. For over two decades, it was postulated that these enzymes have an almost absolute preferences for this amino acid, as confirmed by the structural studies^41^, internally quenched substrate kinetics^34^, and structure-activity relationships of inhibitors with various P1 side-chains^42^. The reason for such narrow P1 specificity is that the S1 pocket is formed by Arg179, Arg341 and Gln283 residues (caspase-1 nomenclature); thus, only negatively charged amino acids of the appropriate size and length can fit. However, the global profiling of protein cleavage events in apoptosis models using proteomic approaches demonstrated that some substrates are cleaved at non-aspartate residues^43^,^44^. Therefore, we hypothesized that caspases might also generate peptides with C-terminal Glu residues^45^. Indeed, it has been reported that caspase-3 cleaves myosin light chain 3 between Glu and Gly (-DFVE/GLRV- sequence) in failing cardiac myocytes^28^. This is the only caspase-3 “Glu” cleavage that has been deposited in the MEROPS databank to date^5^. Moreover, among the other apoptotic caspases, only two “Glu” cleavage sites have been deposited for caspase-9^46^,^47^. To re-examine this phenomenon, we designed and synthesized two internally quenched fluorogenic substrate libraries containing Asp or Glu at P1 and also assessed the role of the P1′ residue in the differences in the cleavage efficiency for Asp versus Glu. Considering the differences in the caspase specificity profiles, we selected the LEHD sequence for initiators (caspase-8, caspase-9, and caspase-10) and DEVD for executioners (caspase-3, caspase-6, and caspase-7). Each library contained either Asp or Glu at P1; thus, 80 individual substrates were synthesized. The general architecture of this library is presented in Fig. 7.

**Figure 8:**
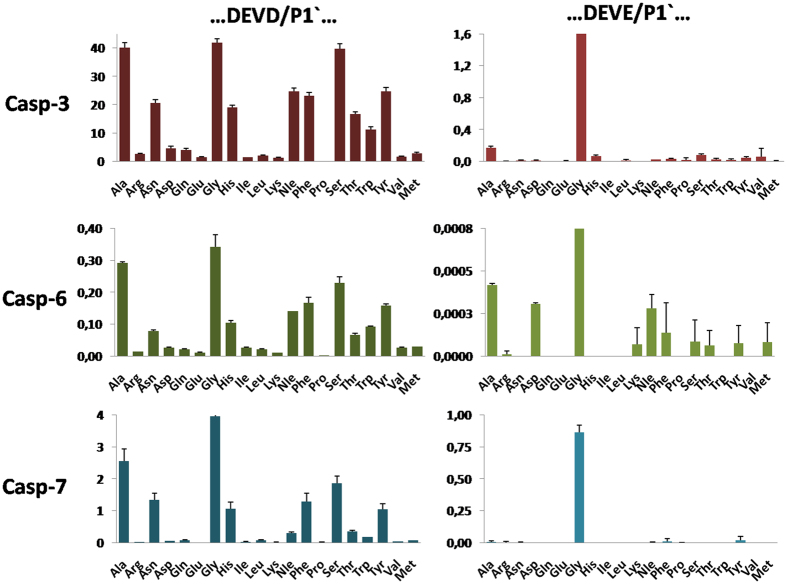
P1′ substrate specificity profiles of caspase-3, caspase-6, and caspase-7, as determined using two internally quenched fluorogenic substrate libraries containing either Asp or Glu at P1 (ACC-GDEV(D/E)-XKK(DNP)G-NH_2_). The library concentration was 2 μM. The x-axis represents amino acids using the standard three-letter code, and the y-axis shows the substrate hydrolysis rate expressed as RFU/s/10 nM caspase (relative fluorescence units per second per 10 nM of caspase).

Executioner caspases (caspase-3, caspase-6, and caspase-7) display a broad P1′ specificity tolerance in the DEVD/X library (the library concentration was 2 μM < K_M_ for the best IQF substrate). In general, all three enzymes prefer small amino acids (Gly, Ala, and Ser); however, aromatic residues (His, Phe, and Tyr) were also well tolerated. However, the P1′ specificity of executioner caspases was markedly different when Glu was present at P1. Caspase-3 has a strong preference for Gly (the second most-favored amino acid is Ala, with approximately 15% of the activity for Gly), caspase-7 accepts only Gly, and caspase-6 displays broader preferences that favor Gly (100%) over Ala, Asp and Nle; however, this enzyme cleaves the DEVE/X substrate very poorly (Fig. 8). This significant S1-S1′ subsite cooperativity might be explained by the poor fit of Glu in the S1 pocket, which forces the peptide substrate to re-adapt to the active site; thus, not all amino acids (other than Gly) can be well accommodated at P1′ in the S1′ pocket. When caspase-3 and caspase-7 were tested on non-preferred substrates containing the initiator caspase consensus (…LEHD/X…), the only amino acid that was tolerated at P1′ was also Gly (Supplemental section). To confirm our findings, we measured the kinetic parameters of six substrates using caspase-3 and caspase-7 (Table 3). Kinetic analysis revealed that caspase-3 and caspase-7 can efficiently cleave peptides after Glu; however, this cleavage occurred almost exclusively when the residue at P1′ was Gly. The substrates containing Glu/Ala and Glu/Ser cleavage sites were poorly recognized by the enzymes, confirming the observed S1-S1′ cooperativity. Moreover, the replacement of Asp with Glu in the DEVX/G substrate resulted in 9- and 3-fold decreases in the activity of caspase-3 and -7, respectively.

**Figure 9:**
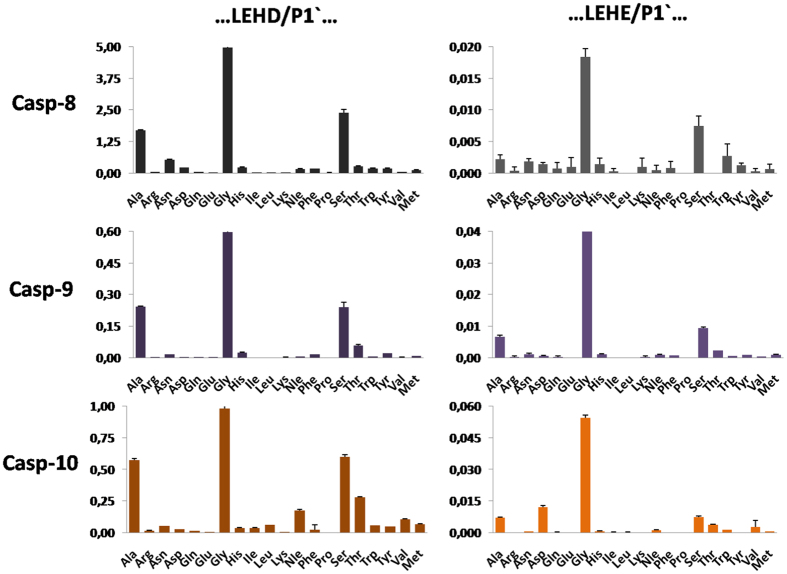
P1′ substrate specificity profiles of caspase-8, caspase-9, and caspase-10, as determined using two internally quenched fluorogenic substrates libraries containing either Asp or Glu at P1 (ACC-GLEH(D/E)-XKK(DNP)G-NH_2_). The library concentration was 2 μM. The x-axis represents amino acids using the standard three-letter code, and the y-axis shows the substrate hydrolysis rate expressed as RFU/s/10 nM caspase (relative fluorescence units per second per 10 nM of caspase).

Next, in a similar manner, we profiled initiator caspases (caspase-8, caspase-9, and caspase-10) at the P1 and P1′ positions (the library concentration was 2 μM) (Fig. 9). When we used the LEHD/X library, we found that all three enzymes displayed very similar preferences at the S1′ pocket, preferring Gly (100% activity), Ala (approximately 40–60% activity), Ser (approximately 40–60% activity), and Thr (up to 20–30% activity). Other amino acids were only poorly or not recognized. When we used the second library (LEHE/X), we observed that caspase-9 and caspase-10 were able to cleave the substrates, whereas caspase-8 displayed only residual activity for substrates containing Glu at P1. Interestingly, unlike the situation for caspase-3 and caspase-7, the P1′ specificity profiles of caspase-8, caspase-9, and caspase-10 do not strongly depend on the presence of Asp or Glu at P1. When these enzymes were tested on non-preferred substrates containing the initiator caspase consensus sequence (…DEVD/X…), caspase-10 exhibited a broader P1′ specificity and preferred the small residues Gly, Ala, and Ser as well as the aliphatic residues Val, Ile, Leu, and Nle. By comparing the results obtained with the LEHD/X and DEVD/X libraries, we demonstrated that the S2 and S1′ pockets were cooperative and that this cooperativity was important for substrate hydrolysis (Supplemental section). In the next step, we measured the kinetic parameters of six substrates in reactions with caspase-8, caspase-9, and caspase-10 (Table 4). An analysis of the k_cat_/K_M_ values showed that the presence of Glu at P1 of the substrate almost prevents caspase-8 cleavage (activity was decreased 500-fold when LEHD/G  → LEHE/G) and limits caspase-9 and caspase-10 cleavage (activity was decreased 7- and 15-fold, respectively, for P1′ Gly substrates). Moreover, a separate analysis of K_M_ and k_cat_ showed that the decrease in activity (P1 D → E) was solely due to k_cat_ because the K_M_ values for caspase-9 and caspase-10 for all six substrates were almost identical (Supplemental section). This finding supports the hypothesis that for the initiator caspases, S1-S1′ cooperativity is not important for binding affinity but still has a considerable influence on the substrate cleavage rate and enzyme turnover.

## Discussion

**Figure 10:**
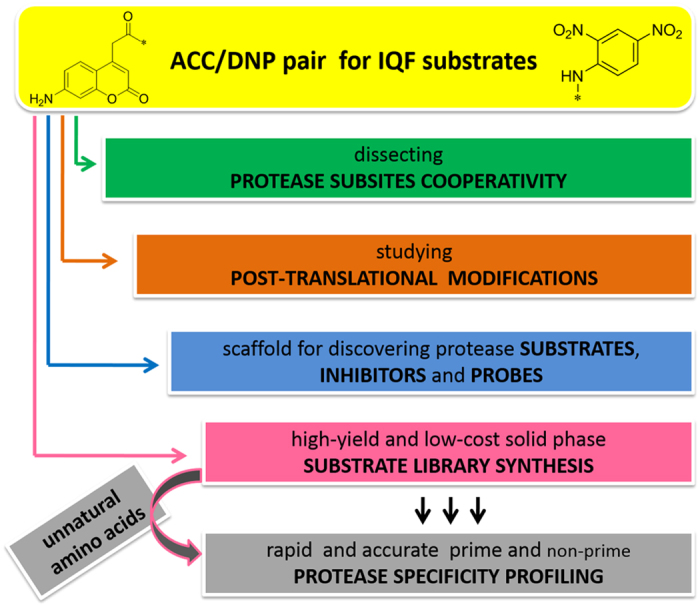
A general outline of the possible applications of ACC/DNP-containing IQF substrates.

IQF substrates are excellent tools to quantitatively define the specificity of proteases in both non-prime and prime regions^34^,^48^,^49^ and afford the opportunity to develop substrate-based markers^50^ or potent inhibitors^51^,^52^ (Fig. 10). Because the process of screening substrates against proteases can be time consuming, there is a need to develop relatively inexpensive new donor/acceptor pairs that provide sensitivity and have reliable synthesis protocols. The ACC/DNP pair is “a rational compromise” because ACC exhibits significantly higher (approximately 10-fold) assay sensitivity compared to that of the commonly used MCA group, and the DNP moiety is far less expensive and less hydrophobic than other quenchers (*e.g.,* Dabcyl). As a proof of principle, we have demonstrated that IQF substrates containing the ACC/DNP pair can be used to study protease preferences for post-translationally modified peptides. We hypothesized that a detailed kinetic analysis of synthetic substrates containing modified amino acids could be considered as an additional validation step to improve the quality of mass spectrometry-based proteomics results. We also postulate that internally quenched substrates can also be used to study other post-translational modifications that occur in natural protease substrates (Fig. 10). A good example for future study would be phosphorylated amino acids, which have been found to influence caspase activity^53^, human plasma and tissue kallikreins activities^54^, as well as hydrolysis by peptidases of [Phospho-Ser(6)]-bradykinin^55^.

Finally, using a tailored internally quenched fluorogenic substrate library, we were able to re-evaluate the quantitative preference for Asp versus Glu at the P1 position of caspase substrates. Caspase-3, caspase-7, caspase-9, and caspase-10 can indeed cleave peptide substrates after Glu, although with much lower efficiency than for substrates containing Asp. The physiological relevance of these non-optimal cleavage rates when the caspase S1 pocket is occupied by Glu is uncertain. However, from a mechanistic point of view, cleavage after Glu is highly influenced by the nature of the residue at P1′, which must be small to exhibit measurable Glu cleavage. Interestingly, this reduction in specificity is not observed for caspase-9 or caspase-10. However, the overall rate of substrate cleavage by caspase-9 and caspase-10 is substantially lower than that by other caspases, leading us to speculate that non-optimal cleavage leads to a broadening of the tolerance for Gly at P1′. This speculation is consistent with previous findings noting that the discrimination for Asp over Glu decreases as the catalytic efficiency of the Drosophila caspase Dronc for substrates decreases^56^. In other words, our data are consistent with the notion that efficient catalysis requires Asp, but decreases in the catalytic efficiency due to non-optimal interactions at other protease subsites reduce the stringency for Asp, allowing Glu to be tolerated. Consequently, we agree that the considerable ability of caspases to cleave peptides after Glu must be considered when analyzing cleavage events in apoptotic cell-based experiments.

## Methods

### Enzymes

The expression and purification of caspase-3, caspase-6, caspase-7, caspase-8, caspase-9, caspase-10, legumain, MMP2 (TIMP2 free) and MMP9 has been described elsewhere^57^,^58^,^59^,^60^, Human neutrophil elastase (HNE) was purchased from Biocentrum (Krakow, Poland). Trypsin was purchased from Sigma Aldrich (Poznan, Poland).

### Chemicals and reagents

All reagents were purchased from commercial sources and used without further purification. The detailed information for this section can be found in supplemental section.

### Substrate design principles

The substrates for caspases, MMPs, legumain, trypsin, and elastase were based on the sequence principles described in the Results section. For each enzyme, we synthesized two substrates, which both had a quencher (DNP moiety) attached to a lysine residue. To compare ACC to MCA, the substrates differed in the nature of the fluorophore used.

### Substrate synthesis

All internally quenched fluorogenic substrates were prepared manually by solid phase synthesis. The detailed description of the IQF substrate synthesis can be found in the supplemental section.

### Substrate kinetics

All kinetic measurements were performed using a Spectra Max Gemini XPS spectrofluorimeter (Molecular Devices) at 37 °C. For the substrates with the new donor acceptor pair, ACC/DNP, the excitation wavelength was 355 nm, and the emission wavelength was 460 nm; for MCA/DNP, the corresponding values were 325 and 420 nm, respectively. The substrate concentrations were varied, and the enzyme concentration was kept constant in each assay; the total reaction volume was 100 μl. Measurements were conducted for 30 min, and only the linear part of the progress curve was used to determine the reaction velocity. The measurements were repeated, and the data presented are the average of at least three measurements. The catalytic parameters k_cat_ and K_M_ were established using GraphPad Prism software to directly fit the initial velocities and initial substrate concentrations to the Michaelis-Menten equation using non-linear regression analysis.

#### Caspases

Caspase assays were performed in a buffer containing 20 mM Pipes, 0.1 M NaCl, 1 mM EDTA, 10 mM DTT, and 10% (w/v) sucrose at pH 7.2 to 7.4^61^. The buffer used for caspase-8, caspase-9 and caspase-10 was supplemented with 1 M sodium citrate^62^. Each caspase was incubated with assay buffer for 30 min at 37 °C before the substrates were added. For caspase-3, the concentration of substrates containing ACC or MCA on the measurement plate ranged from 1 to 50 μM, and the enzyme concentration ranged from 1 to 3 nM. For caspase-7, the concentration of substrates containing ACC or MCA on the measurement plate ranged from 1 to 50 μM, and the enzyme concentration ranged from 10 to 30 nM. For caspase-8, the concentration of a substrates containing ACC or MCA on the measurement plate ranged from 4 to 80 μM, and the enzyme concentration ranged from 20 to 50 nM.

#### Elastase

Assays with neutrophil elastase were performed in a buffer containing 0.1 M HEPES and 0.5 M NaCl at pH 7.5^9^. The enzyme was incubated in assay buffer for 10 min at 37 °C prior to the substrate addition and the measurement. For elastase, the concentration of substrates containing ACC ranged from 0.3 to 5 μM, and the enzyme concentration was 0.344 nM. The concentration of substrates containing MCA was ranged from 0.3 to 5 μM, and the enzyme concentration was 1.4 nM.

#### MMP2 and MMP9

The MMP2 proenzyme was activated with 2 mM 4-aminophenylmercuric acetate (APMA) in 50 mM Tris buffer containing 150 mM NaCl and 5 mM CaCl_2_ for 1 h at 25 °C^60^. The MMP9 proenzyme was activated autocatalytically for 2 h in assay buffer at 25 °C^58^. Assays involving MMP2 and MMP9 were performed in a buffer containing 0.1 M Tris-HCl, 0.1 M NaCl, and 10 mM CaCl_2_ at pH 7.5^58^,^60^. Enzymes were incubated with assay buffer for 40 min at 37 °C prior to the substrate addition and the measurement. For MMP2, the concentration of substrates containing ACC ranged from 3.5 to 60 μM, and the enzyme concentration was 1.25 nM. The concentration of substrates containing MCA was ranged from 5.85 to 100 μM, and the enzyme concentration was 3.8 nM. For MMP9, the concentration of substrates containing ACC ranged from 1.8 to 30 μM, and the enzyme concentration was 2.4 nM. The concentration of substrates containing MCA ranged from 3.0 to 50 μM, and the enzyme concentration was 7.2 nM.

#### Legumain

Pro-legumain was auto-activated in buffer containing 100 mM sodium acetate, 1 mM EDTA, and 10 mM DTT at pH 4.5 for 2.5 h at 37 °C^63^. Assays involving legumain were performed in buffer containing 40 mM citric acid, 1 mM EDTA, 120 mM Na_2_HPO_4_, and 10 mM DTT at pH 5.8. The enzyme was incubated with the assay buffer for 20 min at 37 °C prior to the substrate addition and the measurement. For legumain, the concentration of substrates containing ACC ranged from 1.46 to 25 μM, and the enzyme concentration was 7.3 nM. The concentration of substrates containing MCA ranged from 1.46 to 25 μM, and the enzyme concentration was 24.5 nM.

#### Trypsin

Trypsin was assayed in a buffer containing 0.05 M Tris, 0.15 M NaCl, and 0.01% Triton X100 at pH 7.6. The enzyme was incubated with the assay buffer for 10 min at 37 °C prior to the substrate addition and the measurement. Eight internally quenched substrates were tested in the assay. The trypsin concentration for substrates containing Arg and Lys at P1 was 0.1 nM; for Arg(Me) substrates, the enzyme concentration was 30 nM; and for all other substrates, the enzyme concentration was 200 nM.

### Design and synthesis of substrate libraries for caspases

To investigate the primary (P1) Asp versus Glu specificity of caspases and to determine whether this specificity is influenced by the nature of the P1′ residue, we synthesized four 20-member individual substrate libraries. For the executioner caspases, caspase-3, caspase-6, and caspase-7, we synthesized ACC-GDEVD/XKK(DNP)-GNH_2_ and ACC-GDEVE/XKK(DNP)-GNH_2_, and for the initiator caspases, caspase-8, caspase-9, and caspase-10, we synthesized ACC-GLEHD/XKK(DNP)-GNH_2_ and ACC-GLEHE/XKK(DNP)-GNH_2_. We decided to use Lys at P2′ in the library substrates because this residue provided higher yields during the synthesis and purification than Val. X represents one of 19 natural amino acids (cysteine was excluded) or norleucine. The P4-P2 sequences were selected based on caspase substrate specificity profiles described elsewhere^64^,^65^. All substrates (4 × 20 = 80) were synthesized manually by solid phase synthesis in a similar manner to that described above.

### Screening of the P1-Asp/Glu substrate libraries

To evaluate the preferences of caspase at the P1′ position, we utilized IQF libraries. We tested the DEVD/P1′ and DEVE/P1′ libraries with caspase-3, caspase-6, and caspase-7, and we tested the LEHD/P1′ and LEHE/P1′ libraries with caspase-8, caspase-9, and caspase-10. All enzymes were screened in the above-described caspase buffers (the buffer for caspase-8, caspase-9, and caspase-10 was also supplemented with 1.0 M sodium citrate). All substrates were tested at a final concentration of 2 μM, and the enzyme concentrations used were as follows: 10 nM for caspase-3; 60 nM for caspase-7 for both the DEVD- and DEVE-libraries; 160 and 800 nM for caspase-6 for the DEVD- and DEVE-libraries, respectively; 45, 90, and 50 nM for the LEHD-library for caspase-8, caspase-9, and caspase-10, respectively, and 160, 270, and 200 nM for the LEHE-library for caspase-8, caspase-9, and caspase-10, respectively.

### IQF library substrate kinetics

To obtain further insight into the caspase P1-P1′ preferences, we determined the kinetic parameters for selected IQF substrates. The kinetic analysis was performed in a similar manner to that described above (Substrate Kinetics). The buffers used for caspase-8, caspase-9, and caspase-10 were supplemented with 1.0 M sodium citrate. The enzyme concentrations were as follows: caspase-3, 6 nM; caspase-7, 25 nM; caspase-8, 30–60 nM; caspase-9, 20–140 nM; and caspase-10, 25–125 nM. When testing initiator caspases, the higher enzyme concentration was used for non-optimal LEHE/X substrates.

## Additional Information

**How to cite this article**: Poreba, M. *et al*. Highly sensitive and adaptable fluorescence-quenched pair discloses the substrate specificity profiles in diverse protease families. *Sci. Rep.* **7**, 43135; doi: 10.1038/srep43135 (2017).

**Publisher's note:** Springer Nature remains neutral with regard to jurisdictional claims in published maps and institutional affiliations.

## Supplementary Material

Supplemental Section

## Figures and Tables

**Figure 1:**
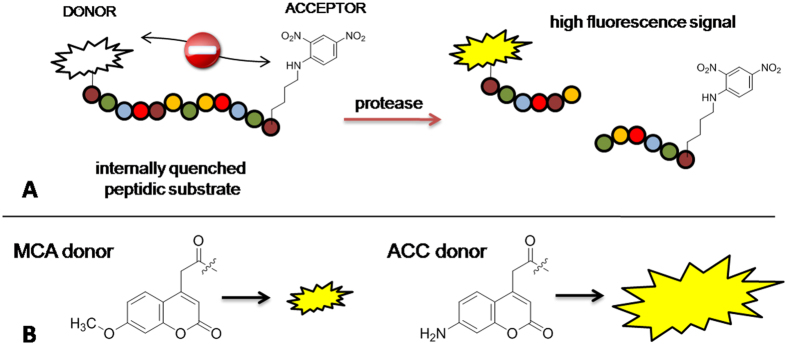
(**A**) Schematic representation of the use of IQF substrates in protease assays. (**B**) Structures of two donor molecules: ACC and MCA. Preliminary experiments allowed us to propose that ACC has a higher quantum yield; thus, substrates containing this donor moiety are more protease sensitive. ACC-Lys(DNP) is a new donor-acceptor pair for use in protease assays.

**Table 1:** Kinetic parameters of four internally quenched substrates with seven proteases of various catalytic types.

Enzyme and substrate	Substrate containing ACC/Lys(DNP)			Substrate containing MCA/Lys(DNP)		
	k_cat_, s^−1^	K_M_, μM	k_cat_/K_M_, M^−1^s^−1^	k_cat_, s^−1^	K_M_, μM	k_cat_/K_M_, M^−1^s^−1^
**Caspase-3** (1.0/3.0 nM)						
Donor-GDEVD*GVK(DNP)D-NH_2_	7.52	6.74	1 116 000	11.24	4.67	2 359 000
**Caspase-7** (10/30 nM)						
Donor-GDEVD*GVK(DNP)D-NH_2_	0.47	8.36	56 800	0.68	6.17	106 000
**Caspase-8** (20/50 nM)						
Donor-GDEVD*GVK(DNP)D-NH_2_	0.32	11.0	29 400	0.32	11.7	26 300
**Legumain** (7.3/24.5 nM)						
Donor-GPTN*KVK(DNP)R-NH_2_	1.35	9.13	149 000	1.09	8.11	139 000
**Elastase** (0.34/1.4 nM)						
Donor-GAEPV*SLK(DNP)L-NH_2_	2.44	1.22	2 006 000	2.58	2.27	1 147 219
**MMP2** (1.25/3.8 nM)						
Donor-GPLG*LK(DNP)AR-NH_2_	4.26	15.4	278 800	6.24	35.1	179 900
**MMP9** (2.4/7.2 nM)						
Donor-GPLG*LK(DNP)AR-NH_2_	0.69	7.81	89 200	0.958	11.7	82 400

Standard deviations were obtained using at least three independent experiments for each measurement and were less than 10%. For each fluorophore, different enzyme concentrations were used (for example, for caspase-3, 1 nM was used for ACC-containing substrates, and 3 nM was used for MCA-containing substrates).

**Table 2:** The detailed kinetic analysis of eight internally quenched substrates containing different P1 amino acid residues for reactions with trypsin.

ACC-GGLS(P_1_)*IVK(DNP)G-NH_2_ hydrolysis by trypsin			
P_1_ variants	k_cat_, s^−1^	K_M_, μM	k_cat_/K_M_, s^−1^M^−1^
Alanine (negative control)	No measurable hydrolysis		
Arginine	24.1	2.50	9 624 000
Arginine(Me)	0.03	17.0	1 820
Arginine(Me)2 symmetrical	0.000523	8.50	62
Arginine(Me)2 asymmetrical	0.000488	19.4	25
Citrulline	0.000359	90.3	4
Lysine	4.2	2.8	1 500 000
Ornithine	0.00170	21.9	78

The standard deviations were less than 10% for the substrates containing Arg/Lys residues and were less than 15% for the substrates containing post-translational modifications. The trypsin concentrations varied from 0.1 nM to 200 nM depending on the substrate used, as described in the Methods section.

**Table 3:** Kinetic parameters of six internally quenched substrates (ACC-GDEV(D/E)-XKK(DNP)G-NH_2_) measured for caspase-3 and caspase-7.

Substrate	Caspase-3				Caspase-7			
	k_cat_, s^−1^	K_M_, μM	k_cat_/K_M_, M^−1^s^−1^	k_cat_/K_M_, E/D ratio	k_cat_, s^−1^	K_M_, μM	k_cat_/K_M_, M^−1^s^−1^	k_cat_/K_M_, E/D ratio
DEVD/G	5.16	3.17	1629000	—	0.926	4.80	193600	—
DEVD/A	4.69	4.21	1930500	—	1.04	6.29	193590	—
DEVD/S	6.52	4.40	1489800	—	0.795	6.38	124700	—
DEVE/G	0.665	3.77	176700	**0.11**	0.475	7.32	65100	**0.34**
DEVE/A	0.357	15.98	22360	**0.012**	0.0162	16.52	958	**0.005**
DEVE/S	0.0646	7.30	8950	**0.006**	0.0032	10.36	307	**0.0025**

The enzyme concentrations varied from 1 to 100 nM depending on the substrate, as described in the Methods section.

**Table 4:** Kinetic parameters of the six internally quenched substrates (ACC-GLEH(D/E)-XKK(DNP)G-NH_2_) measured for caspase-8, caspase-9 and caspase-10.

Substrate	Caspase-8		Caspase-9		Caspase-10	
	k_cat_/K_M_, M^−1^s^−1^	k_cat_/K_M_, E/D ratio	k_cat_/K_M_, M^−1^s^−1^	k_cat_/K_M_, E/D ratio	k_cat_/K_M_, M^−1^s^−1^	k_cat_/K_M_, E/D ratio
LEHD/G	65380	—	3820	—	6740	—
LEHD/A	17000	—	1420	—	4260	—
LEHD/S	29260	—	1640	—	3920	—
LEHE/G	131	**0.002**	525	**0.137**	441	**0.065**
LEHE/A	16	**0.00094**	87	**0.061**	58	**0.014**
LEHE/S	63	**0.002**	132	**0.08**	52	**0.013**

The enzyme concentrations varied from 10 to 240 nM depending on the substrate, as described in the Methods section.
