# Supplementary material for: Highly sensitive and adaptable fluorescence-quenched pair discloses the substrate specificity profiles in diverse protease families
Source: Sci Rep. 2017 Feb 23;7:43135. doi: 10.1038/srep43135 (PMC5322338; doi:10.1038/srep43135)
Supplement: Supplemental Section [file srep43135-s1.pdf]

SUPPLEMENTARY INFORMATION

Highly sensitive and adaptable fluorescence-quenched pair discloses the substrate specificity profiles in diverse protease families

Marcin Poreba[1,3*], Aleksandra Szalek[1], Wioletta Rut[1], Paulina Kasperkiewicz[1,3], Izabela Rutkowska-Wlodarczyk[2], Scott S. Snipas[3], Yoshifumi Itoh[4], Dusan Turk[5], Boris Turk[5], Christopher M. Overall[6], Leszek Kaczmarek[2], Guy S. Salvesen[3,*], Marcin Drag[3*]

[1]Department of Bioorganic Chemistry, Faculty of Chemistry, Wroclaw University of Technology, Wyb. Wyspianskiego 27, 50-370 Wroclaw, Poland

[2]Laboratory of Neurobiology, Nencki Institute of Experimental Biology, Polish Academy of Sciences, 02-093 Warsaw, Poland

[3]NCI-designated Cancer Center, Sanford-Burnham Prebys Medical Discovery Institute, La Jolla, CA 92037, USA

[4]Kennedy Institute of Rheumatology, University of Oxford, United Kingdom

[5]Department of Biochemistry and Molecular and Structural Biology, Jožef Stefan Institute, SI-1000 Ljubljana, Slovenia

[6]Centre for Blood Research, Department of Oral Biological and Medical Sciences, University of British Columbia, Vancouver, BC V6T 1Z3, Canada

*Correspondence to marcin.poreba@pwr.edu.pl, gsalvesen@sbpdiscovery.org, or marcin.drag@pwr.edu.pl

Materials and procedures:

Chemicals and reagents. All Fmoc-protected amino acids (purity > 99%) were purchased from Iris Biotech GmbH (Marktredwitz, Germany). Fmoc-Rink amide AM polystyrene resin (particle size 200-300 mesh, loading 0.48 mmol/g) for peptide synthesis, diisopropyl carbodiimide (peptide grade) and trifluoroacetic acid (purity 99%) were obtained from Iris Biotech GmbH. *N*-hydroxybenzotriazole (purity > 98%) was purchased from Creosalus (Louisville, USA). Methanol, dichloromethane and diethyl ether (all of analytical purity grade), and phosphorus pentoxide (purity 98%) were obtained from POCh (Gliwice, Poland). *N,N'*-dimethylformamide (peptide grade) and acetonitrile (HPLC gradient grade) were purchased from J.T. Baker (Center Valley, USA). Piperidine (purity > 99%) and triisopropylsilane (purity 99%) were obtained from Sigma Aldrich (Poznan, Poland). All reagents/chemicals listed above were used without additional purification. All synthesized peptide substrates were subjected to HPLC on a Waters M600 solvent delivery module using a semi-preparative Waters Spherisorb S10ODS2 column equipped with a Waters M2489 detector system. The solvent components were as follows: water/0.1% TFA for phase A and acetonitrile/0.1% TFA for phase B. The collected fractions were subjected to analytical HPLC using a Waters Spherisorb S5ODS2 column. Mass spectrometry was conducted to confirm the molecular weights of the purified peptides; to this end, a High-Resolution Mass Spectrometer WATERS LCT Premier XE instrument equipped with Electrospray Ionization (ESI) and Time of Flight (TOF) instruments were used.

Substrate synthesis. The ACC fluorophore was prepared as described previously by Maly *et al.*, (J Org Chem, 2002, 67(3); 910-915) and MCA was synthesized in a similar manner using 3-methoxyphenol and 1,3-acetone dicarboxylic acid as starting materials. For each substrate, 100 mg (0.048 mmol) of Fmoc-Rink Amide AM polystyrene resin with an amine substitution level of 0.48 mmol/g was used. The resin was placed in the cartridge, and anhydrous dichloromethane was added; the mixture was then incubated for 1 h at room temperature with occasional gentle stirring. The resin was then filtered and washed with DMF (3 times). Subsequently, the Fmoc protecting group was removed from the Fmoc resin; 20% piperidine in DMF was the added to the glass reaction vessel, which was then agitated for periods of 5 min, 5 min and 25 min. Each time, the piperidine solution was filtered, and the resin was washed with DMF. The first Fmoc-amino acid (Fmoc-P1-OH) was then added to the

resin in 2.5-fold excess (over the resin load); 0.12 mmol of amino acid was used in the synthesis. The amino acid was first preactivated with HOBt (0.12 mmol) and DICI (0.12 mmol) in DMF in an Eppendorf tube before transfer to the resin. The mixture was agitated for 2 h and then filtered and washed with DMF (3 times). Fmoc-P1 (amino acid)-resin was obtained in this way. In the next step, the Fmoc protecting group was removed using 20% piperidine in DMF as described above. The peptide substrates were then elongated in the same manner to obtain 8 (or 9) amino acids (depending on the substrate). Finally, the fluorophore (donor) was attached to the N-terminus. The fluorophore (ACC or MCA; 0.12 mmol) and HOBt (0.12 mmol) were dissolved in DMF; then, DICI (0.12 mmol) was added to the mixture, and the solution was immediately transferred to the resin. The reaction was carried out for 24 h at room temperature under continuous agitation. The resin was then filtered and washed with DMF (3 times). The reaction was then repeated with the same amount of reagents to increase the efficiency of fluorophore coupling. Next, the Fmoc protecting group was removed from the substrates containing the ACC fluorophore as described above. The substrates containing the MCA fluorophore did not have a protecting group, and this step was therefore omitted. The resins containing all substrates were then washed (in order) with DMF (5 times), dichloromethane (3 times), and methanol (3 times) and were finally dried over P_2O_5 in a desiccator. The peptides were then cleaved from the resin using trifluoroacetic acid:triisopropylsilane:water (v:v:v, 95:2.5:2.5). Two milliliters of the solution was transferred to each glass reaction vessel, and the reactions were incubated for 2 h at room temperature with occasional stirring. The solutions containing the peptide substrates were then collected in Falcon-type tubes. Next, diethyl ether was added to the solutions, and the tubes were placed in a freezer for 30 min. Subsequently, after precipitation, the Falcon-type tubes were centrifuged for 5 min (4.4×10^3 rpm), the supernatants were removed, and diethyl ether was again added to the tubes. After centrifugation, the supernatants were discarded. The crude products were subjected to preparatory HPLC and lyophilized. The purity of the collected fractions was confirmed by analytical HPLC. High-resolution mass spectrometry was applied to confirm the molecular weights of the purified peptides. The final products were dissolved in DMSO to a concentration of 10 mM.

Determination of fluorescent quantum yields (ϕF) for ACC and MCA. The fluorescent quantum yield for the ACC fluorophore was measured by a comparative method according to Thermo Scientific technical note 52019: "Measurement of Fluorescence Quantum Yields" by Michael Allen. As a reference fluorophore the 7-diethylamino-4-methylcoumarin (Coumarin 1) was selected (Sigma Aldrich, D87759). The quantum yields (in various solvents) for this fluorophore was measured by Jones II (*J. Phys. Chem.* **1985**, 89, 294-300). In brief,

serial dilutions (from 200µM to 10µM) of free ACC and Coumarin-1 was made in various solutions (100% ethanol, 50% ethanol, 20% ethanol, and 0% ethanol in water) and their absorbance values at 360 nm were recorded (at 37°C). Next, for each sample the fluorescence spectrum from 385nm to 600nm was measured (excitation 360nm) and the integrated fluorescence intensity was calculated (at 37°C). Subsequently, the integrated fluorescence intensity was plotted against the absorbance at 360nm and for each fluorophore, in each solvent, the slopes were calculated. The quantum yield of ACC was calculated according to the formula (the results are collected in Table S1 and Figure S1):

$$\phi F, ACC = \phi F, coumarin - 1 \times \frac{slope, ACC}{slope, coumarin - 1}$$

The ϕF of free MCA (in various solvents, including water) was measured based on the known MCA ϕF value (0.18) measured in methanol by Farinotti et al. (J. Chromatography, 1983, 269, 81-90). The MCA absorbance at 320nm (in various solvents) was measured (at 37°C) and plotted against the integrated fluorescence intensity values (calculated from fluorescence spectra from 345nm to 600nm, excitation 320nm). The MCA ϕF was then calculated using the equation (where "*n*" is the refractive index of the solvent):

$$\phi F, MCA_{solvent} = \phi F, MCA_{\mathrm{MeOH}} \times \frac{slope, MCA_{solvent}}{slope, MCA_{MeOH}} \times \frac{n^2{}_{solvent}}{n^2{}_{MeOH}}$$

Determination of Förster distance (R_0) for ACC/dnp and MCA/dnp pairs. In order to measure the R_0 distance (in Å) for ACC/dnp and MCA/dnp pairs we used equation *13.5* from "Principles of fluorescence spectroscopy" by Lakowicz (Second Edition):

$$R_0 = 0.211[\kappa^2 n^{-4} Q_D J(\lambda)]^{1/6}$$

where, κ^2 is a factor describing the relative orientation in space of donor and acceptor dipoles (usually assumed to be 2/3), *n* is refractive index of the solvent (1.33 for water), Q_D is the fluorescence quantum yield of donor in the absence of a quencher, and J(λ) is the overlap integral (a degree of spectral overlap between the emission spectrum of donor and the absorption spectrum of a quencher). The J(λ) can be calculated from the equation:

$$J(\lambda) = \frac{\int_0^\infty F_D(\lambda)\varepsilon_A(\lambda)\lambda^4 d\lambda}{\int_0^\infty F_D(\lambda) d\lambda}$$

where the λ is the wavelength, and the ε_A is the extinction coefficient of a quencher. If ε_A is expressed in $M^{-1}cm^{-1}$, then the J(λ) is expressed in $M^{-1}cm^{-1}nm^4$. Since we used an Excel software to calculate the overlap integral, we transformed the equation above into the series form:

$$J(\lambda) = \frac{\sum_{\lambda=300, \lambda\in N}^{600} F_D(\lambda)\varepsilon_A\lambda^4}{\sum_{\lambda=300, \lambda\in N}^{600} F_D(\lambda)}$$

Spectra were recorded from 300nm to 600nm, with 1nm intervals following the above equation.

Solvent, % in water	Free MCA	Free ACC	Coumarin-1
100% MeOH	0.180[a]	n.d.	n.d.
100% EtOH	0.207	0.850	0.73[b]
50% EtOH	0.480	0.835	0.30[b]
20% EtOH	0.609	0.856	0.11[b]
water only	0.718	0.861	0.055[b]

Table S 1 The fluorescence quantum yields measured for ACC and MCA in various solvents. Ref. **“a”** Farinotti et al. (*J. Chromatography*, 1983, 269, 81-90), ref. **“b”** Jones II (*J. Phys. Chem.* 1985, 89, 294-300).

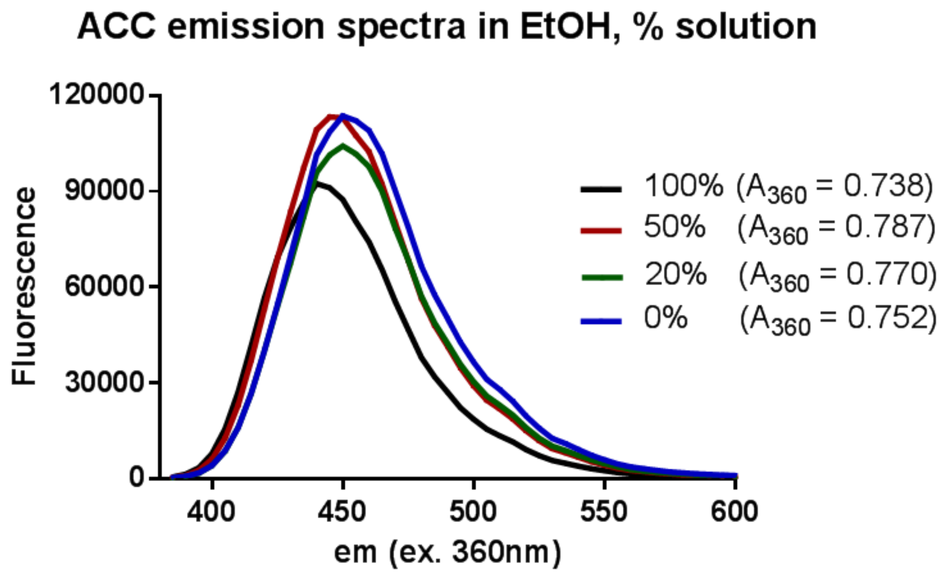

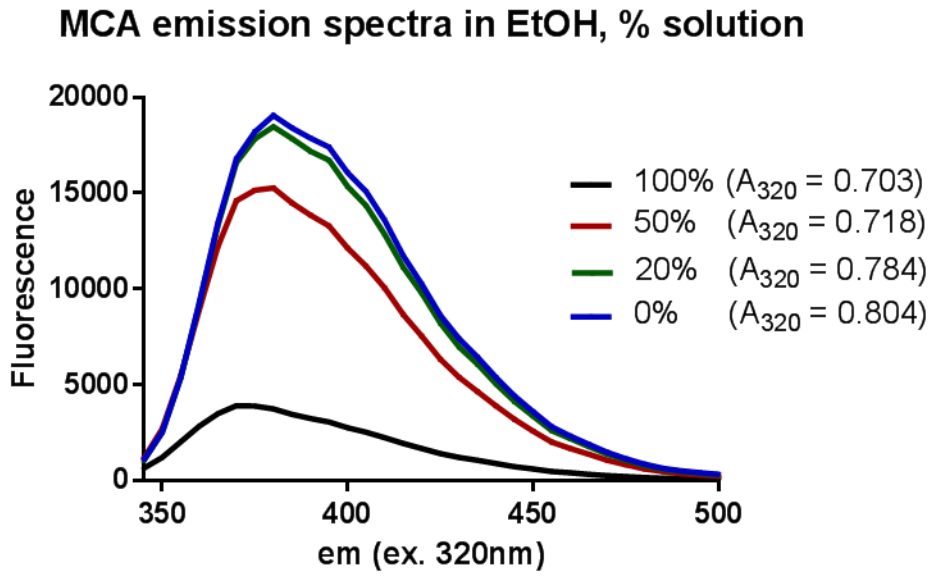

Figure S 1 The emission spectra of ACC and MCA fluorophores measured in various solvents. The data indicate that ACC has comparable quantum yield in all four solvents, whereas MCA has the highest quantum yield is “less ethanol” solvents.

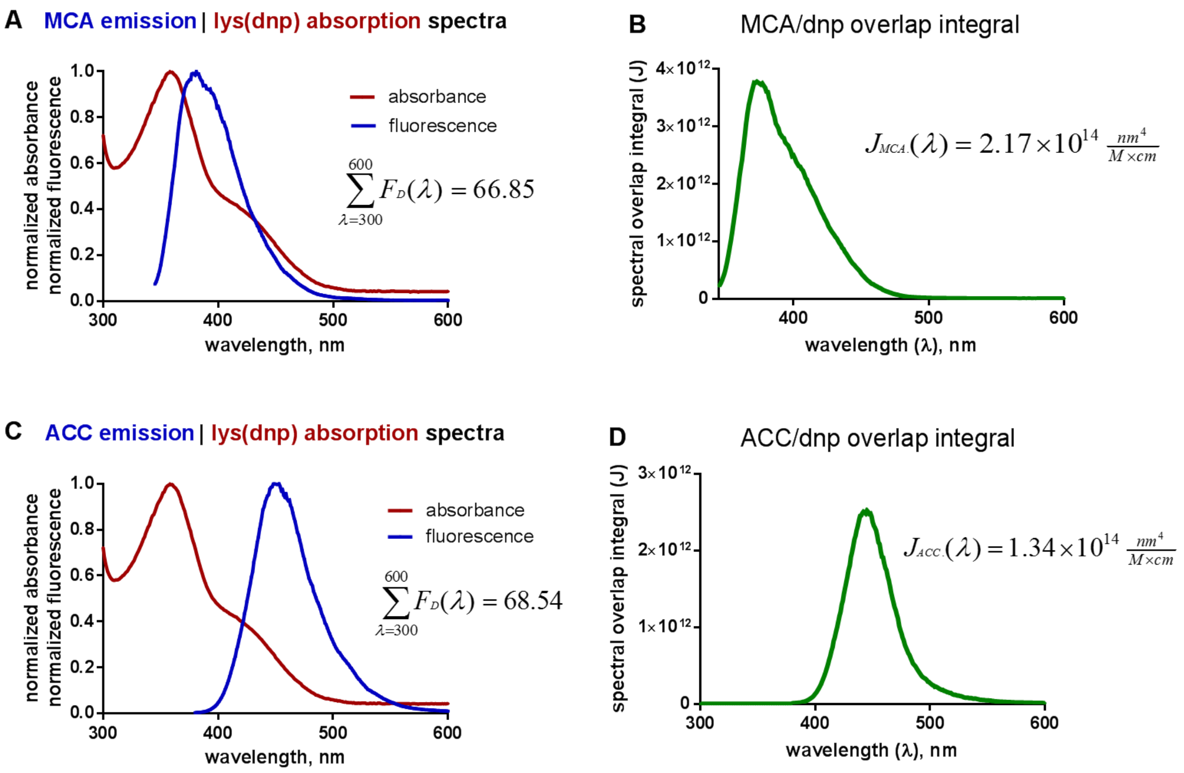

Figure S 2 The calculation of spectra overlap integral J(λ) for ACC/dnp and MCA/dnp pairs. **A** and **C** are superposition of MCA/lys(dnp) and ACC/lys(dnp) spectra, respectively. For both fluorescence spectra cumulative normalization factor ($F_D(\lambda)$) was calculated. B and D are spectra overlap integral plots calculated from A and C plots, respectively.

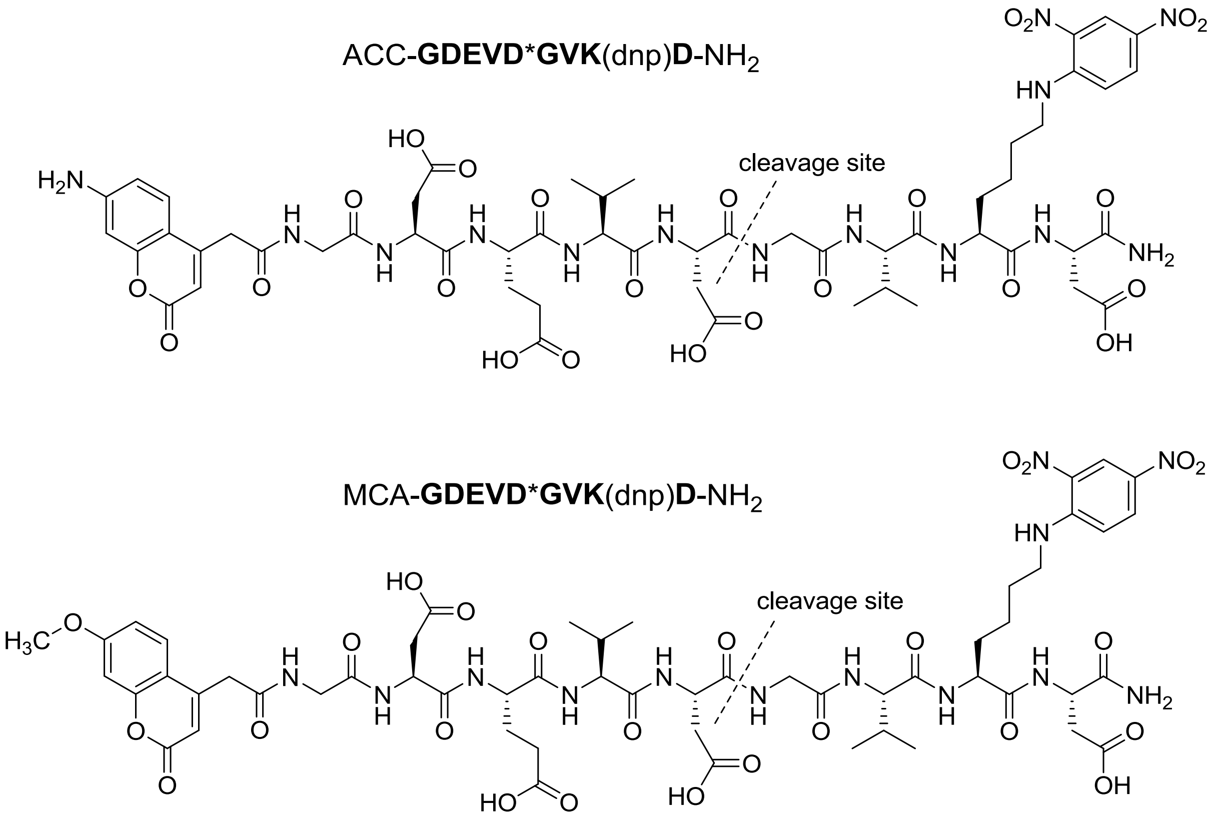

Figure S 3 The structures of two internally quenched (ACC and MCA-labeled) substrates for the investigation of caspase -3, -7, and -8.

substrate / peptide fragment	[M + H+] expected	[M + H+] found
ACC-GDEVD*GVK(dnp)D-NH2	1299.4766	1299.4855
ACC-GDEVD-OH	735.2473	735.2679 (casp-3)
NH2-GVK(dnp)D-NH2	583.2476	583.2469 (casp-3)<br>583.2423 (casp-7)<br>583.2486 (casp-8)
MCA-GDEVD*GVK(dnp)D-NH2	1314.4762	1314.4669
MCA-GDEVD-OH	772.2289 (+Na+)	772.2294 (+Na+) (casp-3)
NH2-GVK(dnp)D-NH2	583.2476	583.2222 (casp-3)<br>583.2487 (casp-7)<br>583.2489 (casp-8)

Table S 2 The HR-MS analysis of the ACC- and MCA-labeled internally quenched substrates and their N-terminal and C-terminal cleavage fragments produced by caspases-3, -7 and -8.

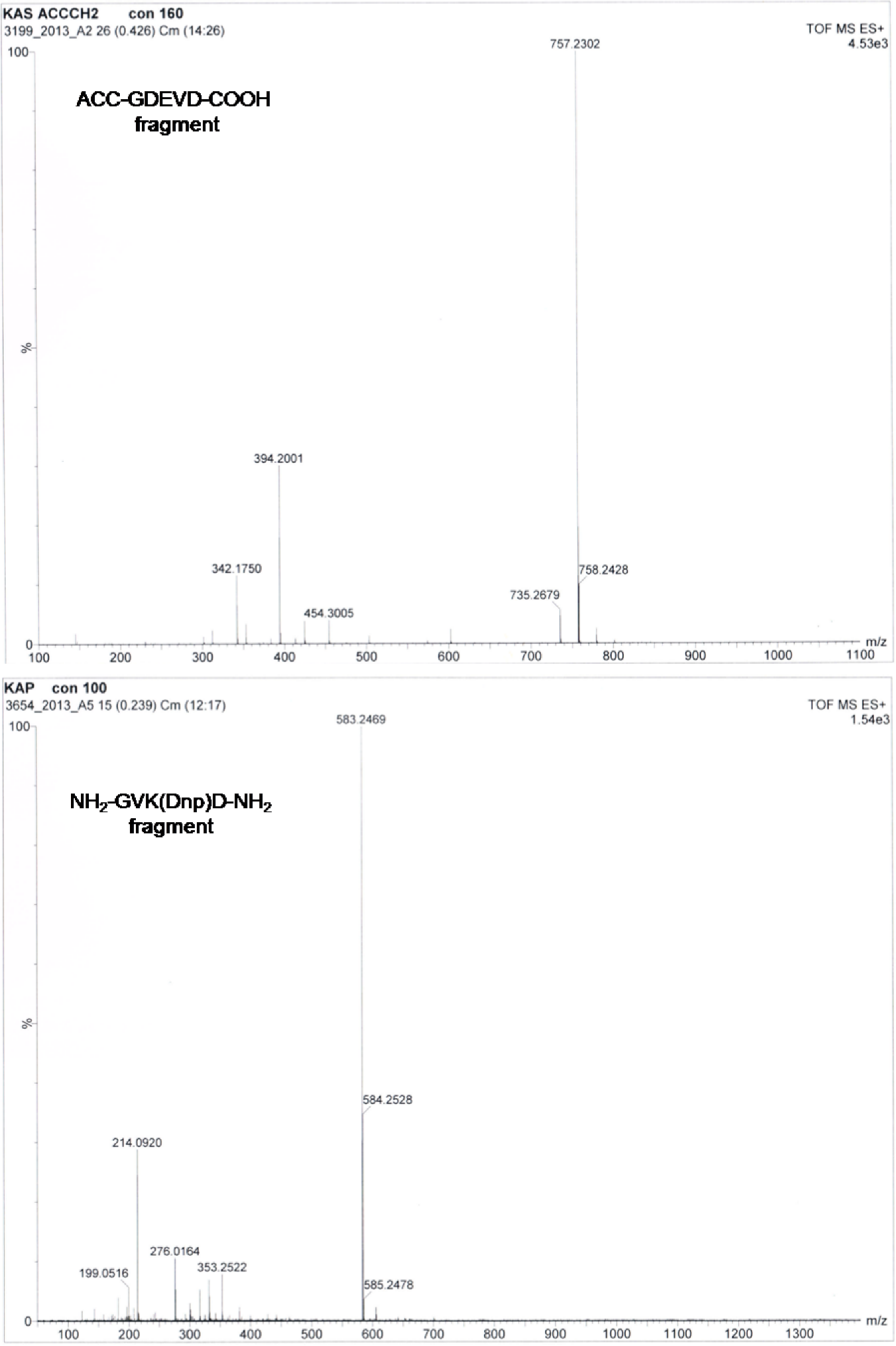

Figure S 4 The HR-MS analysis of the ACC-GDEVD/GVK(dnp)D-NH_2 cleavage fragments: N-terminal ACC-GDEVD-COOH and C-terminal NH_2-GVK(Dnp)D-NH_2 produced by caspase-3.

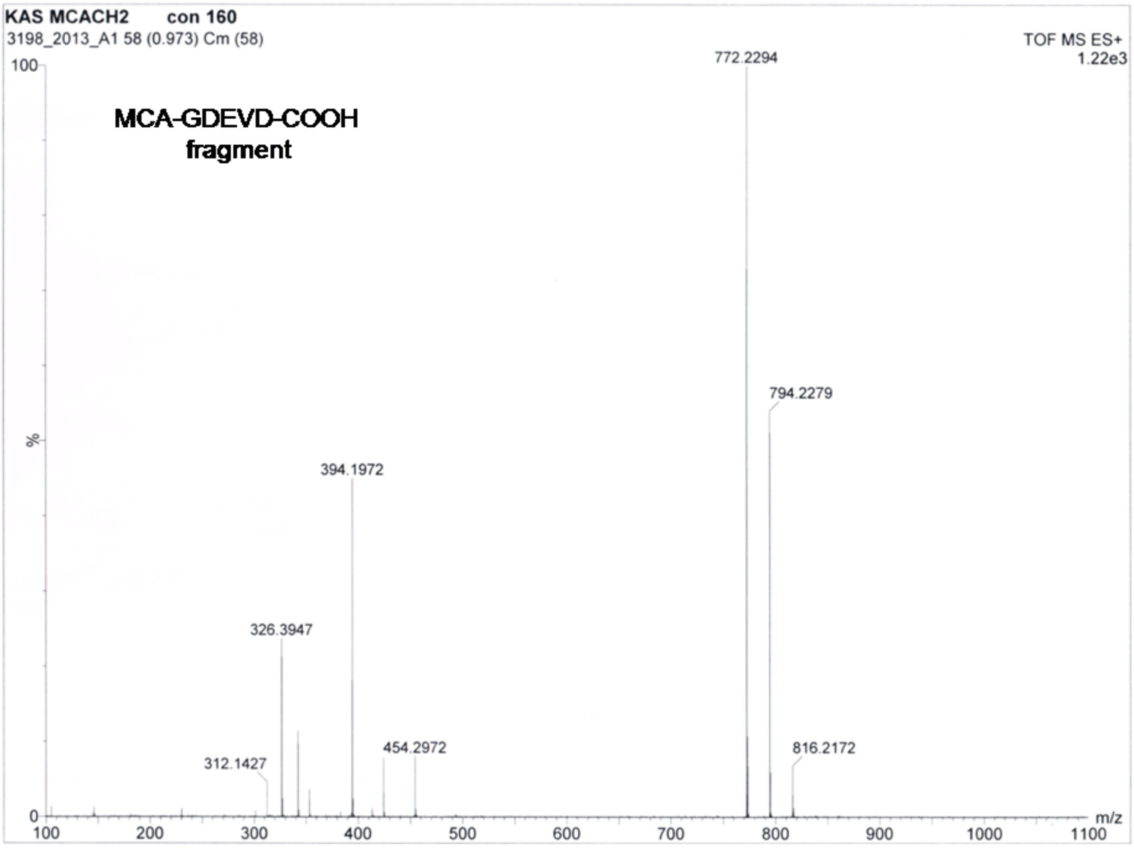

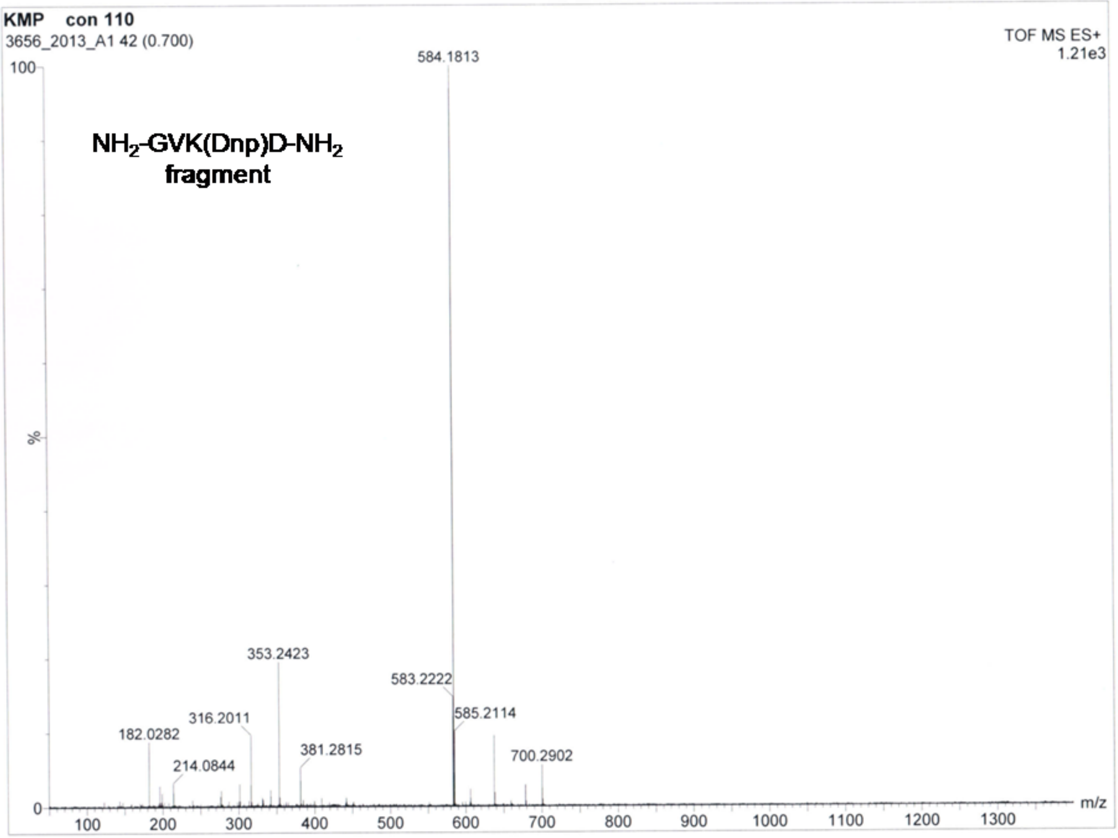

Figure S 5 The HR-MS analysis of the MCA-GDEVD/GVK(dnp)D-NH_2 cleavage fragments: N-terminal MCA-GDEVD-COOH and C-terminal NH_2-GVK(Dnp)D-NH_2 produced by caspase-3.

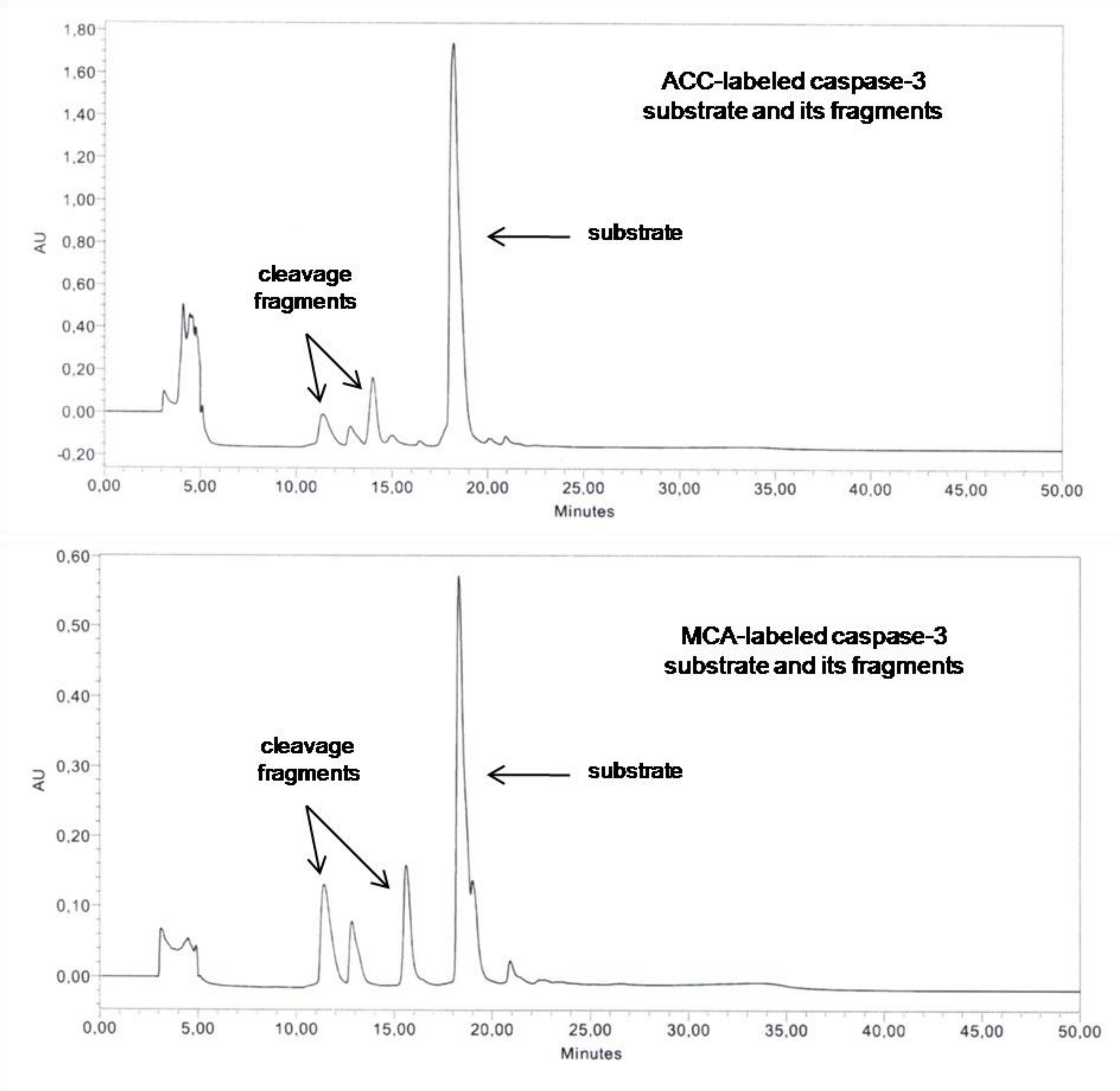

Figure S 6 The HPLC analysis of ACC- and MCA-labeled internally quenched substrates (ACC/MCA-GDEVDGVK(dnp)D-NH_2) for caspase-3. The cleavage fragments indicated in the figure were separated by semi-preparative HPLC and subjected to HR-MS analysis to confirm the caspase-3 cleavage site.

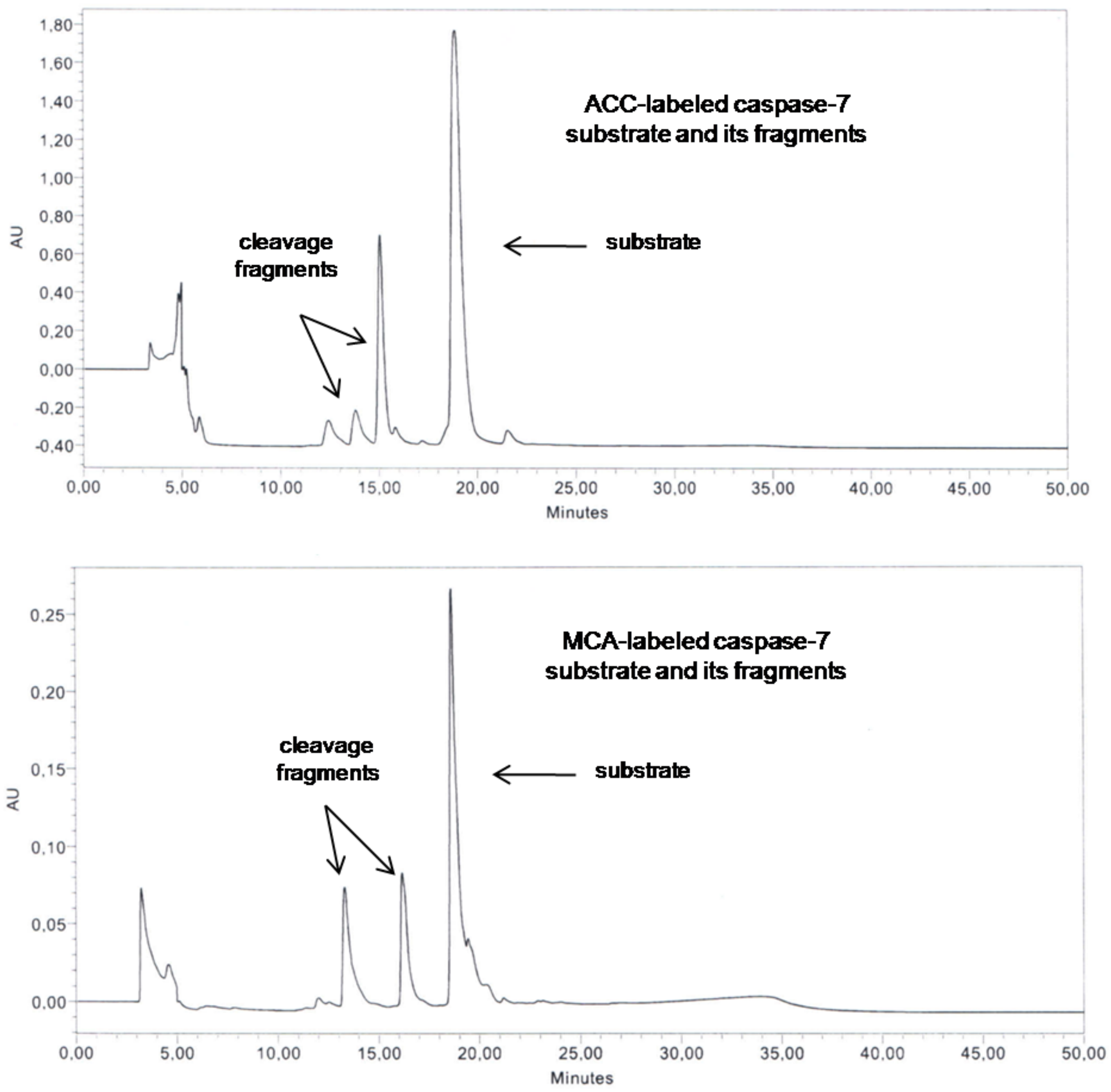

Figure S 7 The HPLC analysis of ACC- and MCA-labeled internally quenched substrates (ACC/MCA-GDEVDGVK(dnp)D-NH_2) for caspase-7. The cleavage fragments indicated in the figure were separated by semi-preparative HPLC and subjected to HR-MS analysis to confirm the caspase-7 cleavage site.

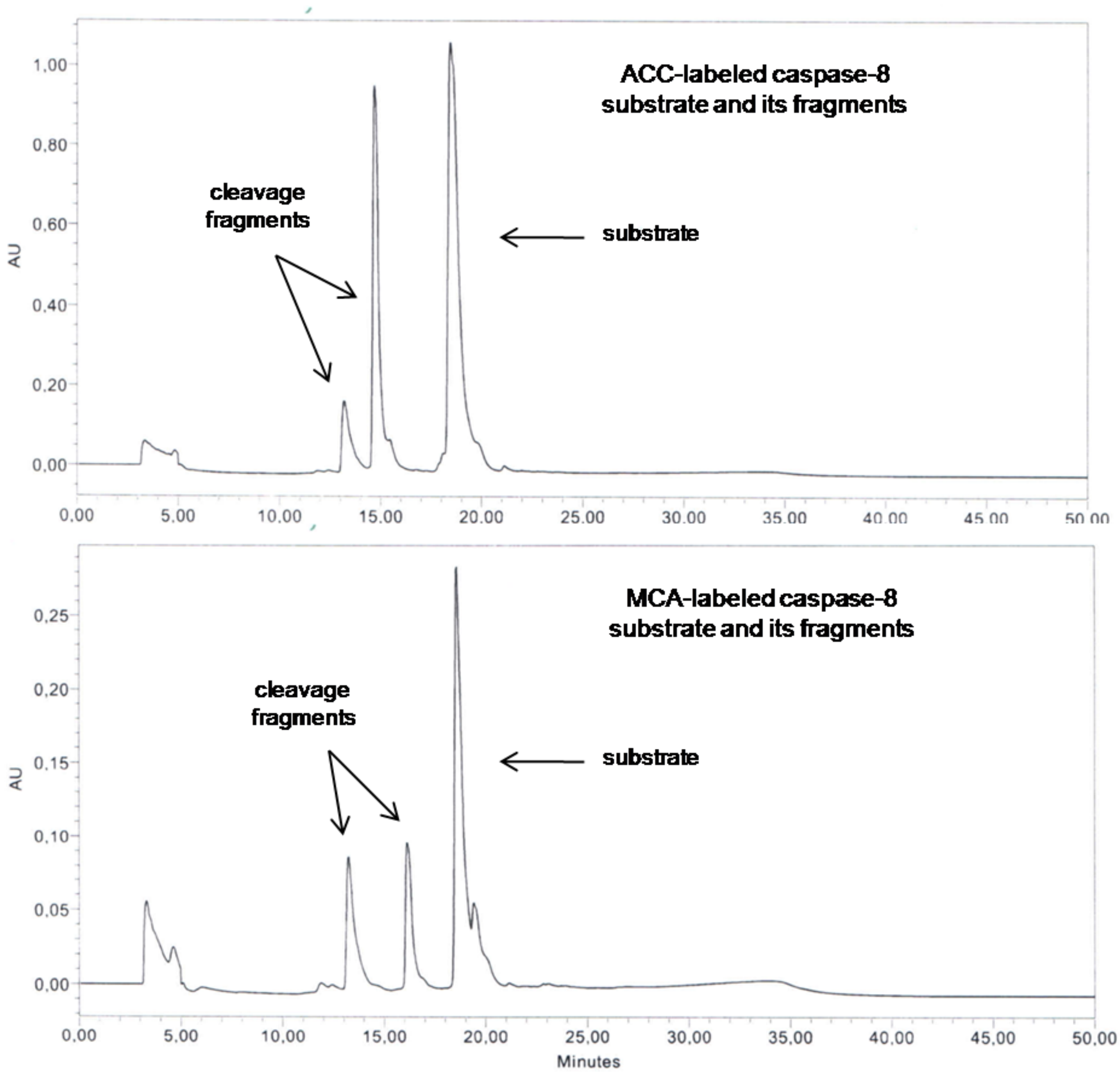

Figure S 8 The HPLC analysis of ACC- and MCA-labeled internally quenched substrates (ACC/MCA-GDEVDGVK(dnp)D-NH_2) for caspase-8. The cleavage fragments indicated in the figure were separated by semi-preparative HPLC and subjected to HR-MS analysis to confirm the caspase-8 cleavage site.

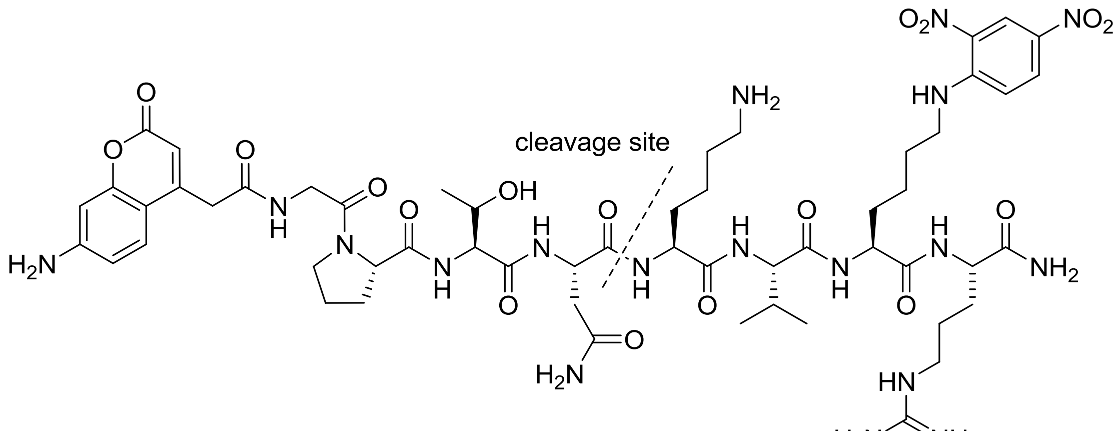

ACC-**GPTN***KVK**(dnp)**R**-NH_2

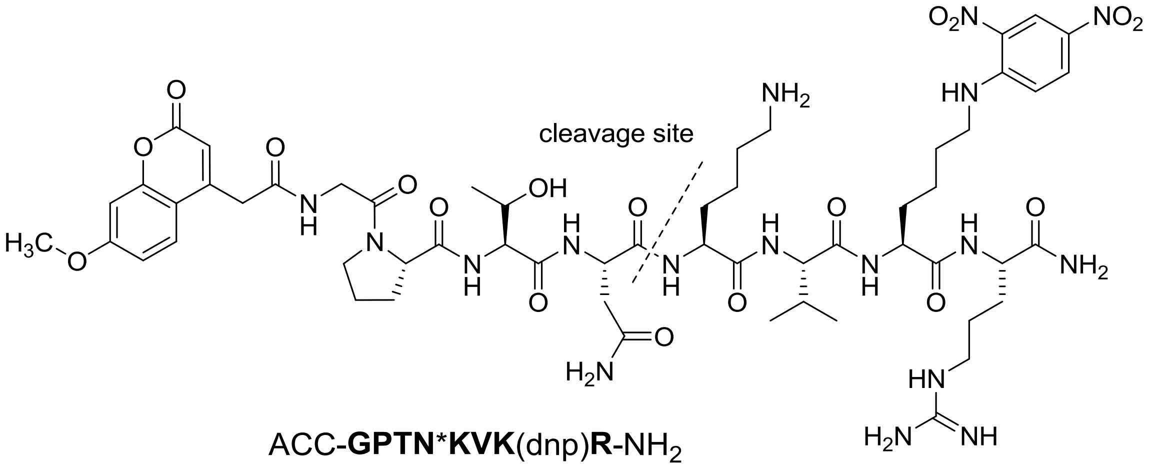

ACC-**GPTN***KVK**(dnp)**R**-NH_2

Figure S 9 The structures of two internally quenched (ACC and MCA-labeled) substrates for the investigation of human legumain.

substrate / peptide fragment	[M + H+] expected	[M + H+] found
ACC-GPTN*KVK(dnp)R-NH_2	1265.6027	1265.6088
ACC-GPTN-OH	589.2258	589.2271
NH_2-KVK(dnp)R-NH_2	695.3953	695.3961
MCA-GPTN*KVK(dnp)R-NH_2	1280.6024	1280.6159
MCA-GPTN-OH	626.2074 (+Na^+)	626.2083 (+Na^+)
NH_2-KVK(dnp)R-NH_2	695.3953	695.3957

Table S 3 The HR-MS analysis of the ACC- and MCA-labeled internally quenched substrates and their N-terminal and C-terminal cleavage fragments produced by human legumain.

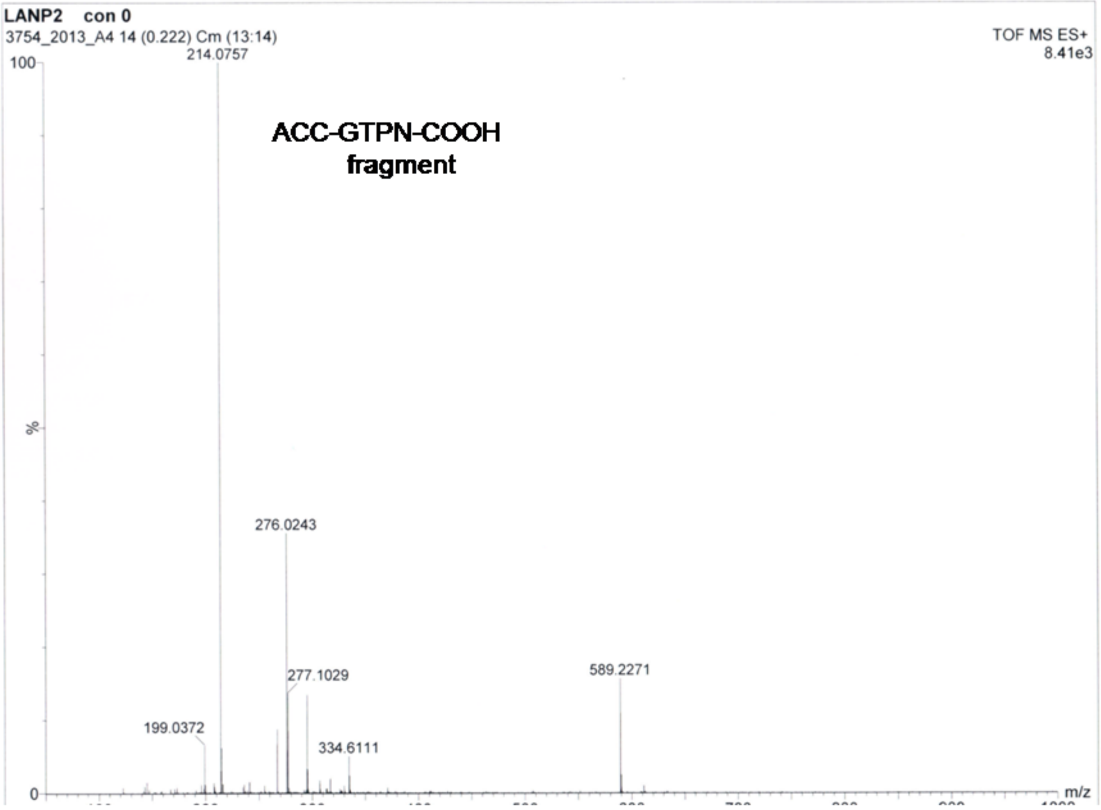

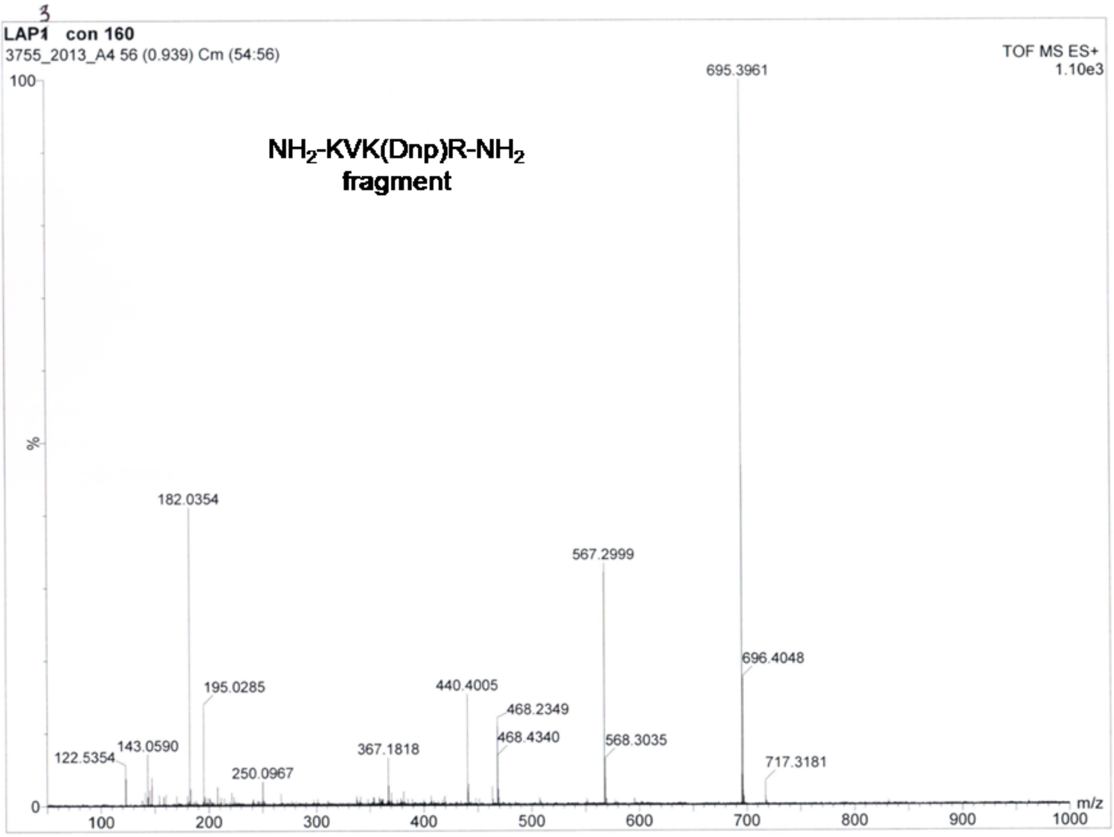

Figure S 10 The HR-MS analysis of the ACC-GTPN/KVK(dnp)R-NH_2 cleavage fragments: N-terminal ACC-GTPN-COOH and C-terminal NH_2-KVK(Dnp)R-NH_2 produced by human legumain.

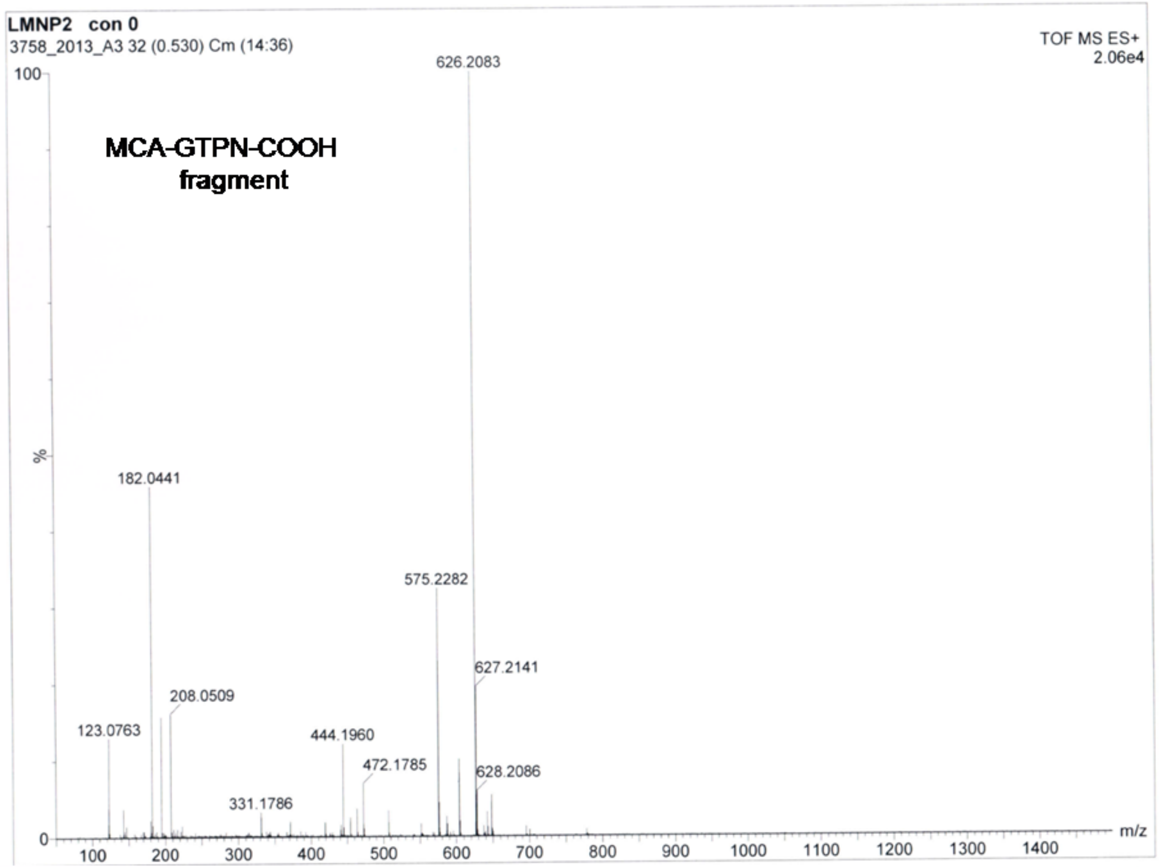

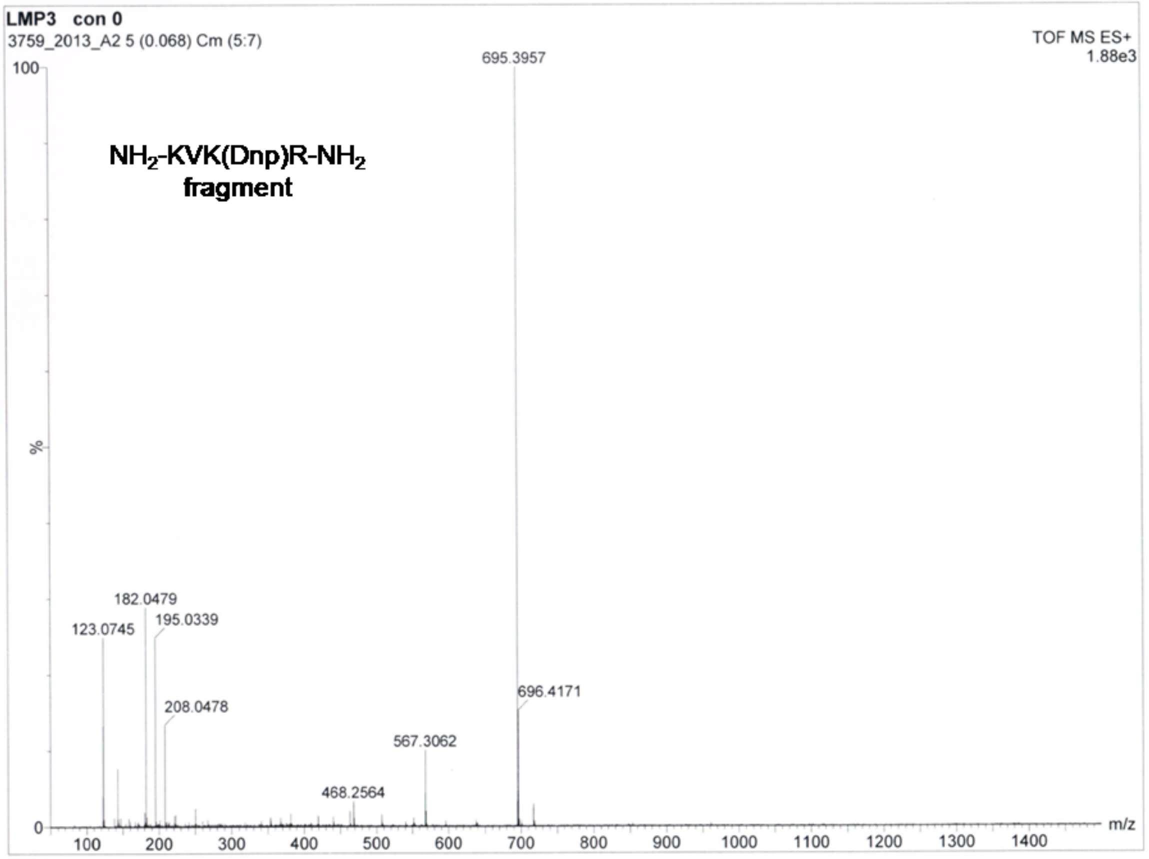

Figure S 11 The HR-MS analysis of the MCA-GTPN/KVK(dnp)R-NH_2 cleavage fragments: N-terminal MCA-GTPN-COOH and C-terminal NH_2-KVK(Dnp)R-NH_2 produced by human legumain.

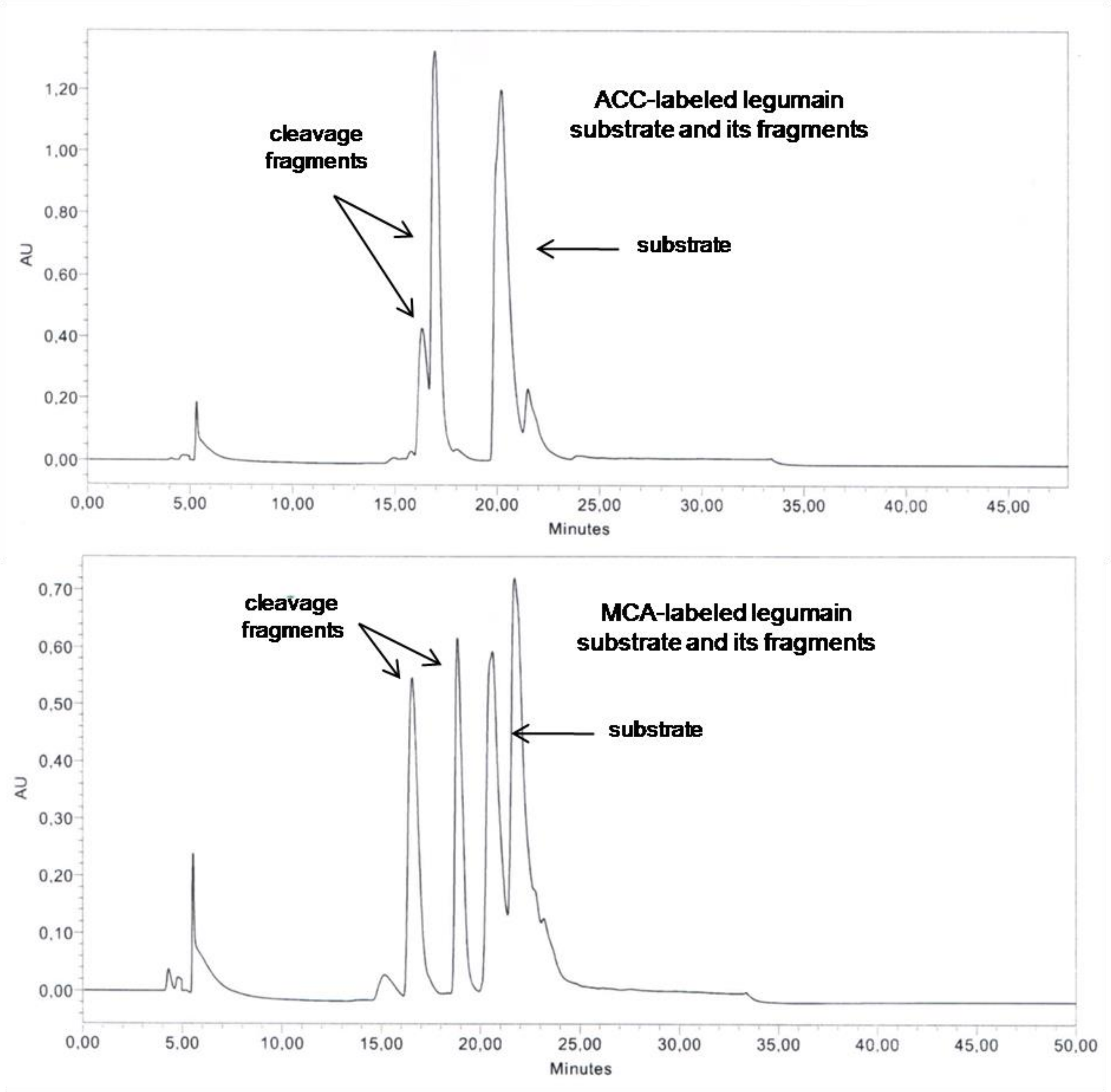

Figure S 12 The HPLC analysis of ACC- and MCA-labeled internally quenched substrates (ACC/MCA-GTPNKVK(dnp)R-NH_2) for legumain. The cleavage fragments indicated in the figure were separated by semi-preparative HPLC and subjected to HR-MS analysis to confirm the legumain cleavage site.

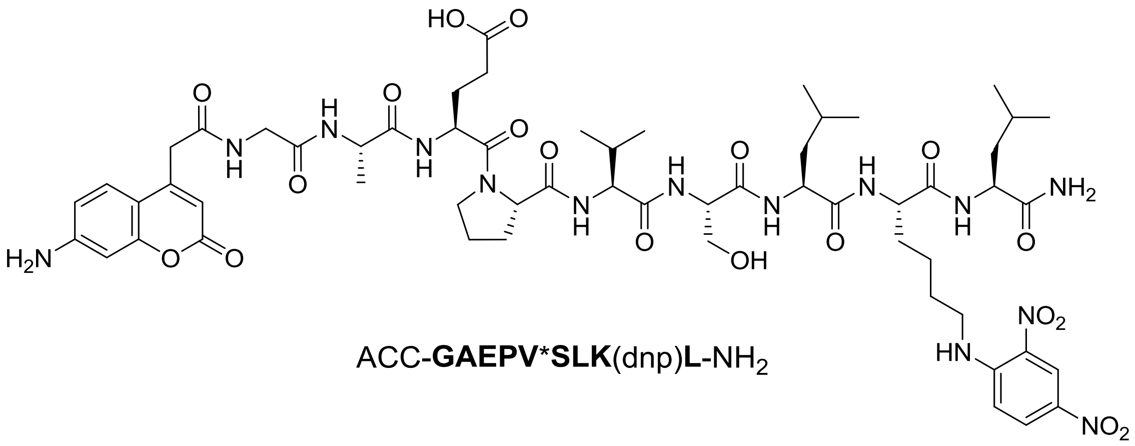

ACC-**GAEPV***SLK**(dnp)**L**-NH_2

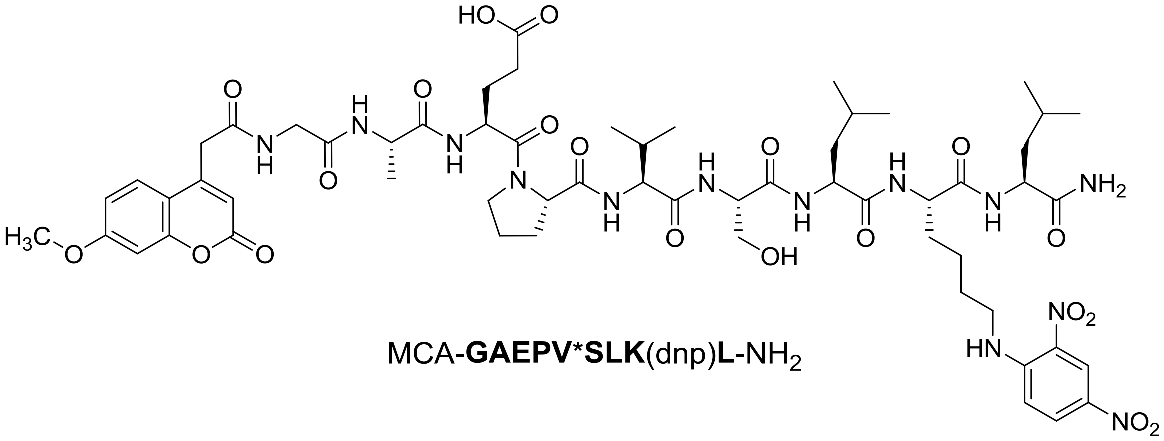

MCA-**GAEPV***SLK**(dnp)**L**-NH_2

Figure S 13 The structures of two internally quenched (ACC and MCA-labeled) substrates for the investigation of human neutrophil elastase.

substrate / peptide fragment	[M + H^+] expected	[M + H^+] found
ACC-GAEPV*SLK(dnp)L-NH_2	1279.5959	1279.6224
ACC-GAEPV-OH	673.2833	673.2818
NH_2-SLK(dnp)L-NH_2	647.3129 (+Na^+)	647.3550 (+Na^+)
MCA-GAEPV*SLK(dnp)L-NH_2	1294.5956	1294.6248
MCA-GAEPV-OH	688.2830	688.3044
NH_2-SLK(dnp)L-NH_2	625.3310	625.3399

Table S 4 The HR-MS analysis of the ACC- and MCA-labeled internally quenched substrates and their N-terminal and C-terminal cleavage fragments produced by human neutrophil elastase.

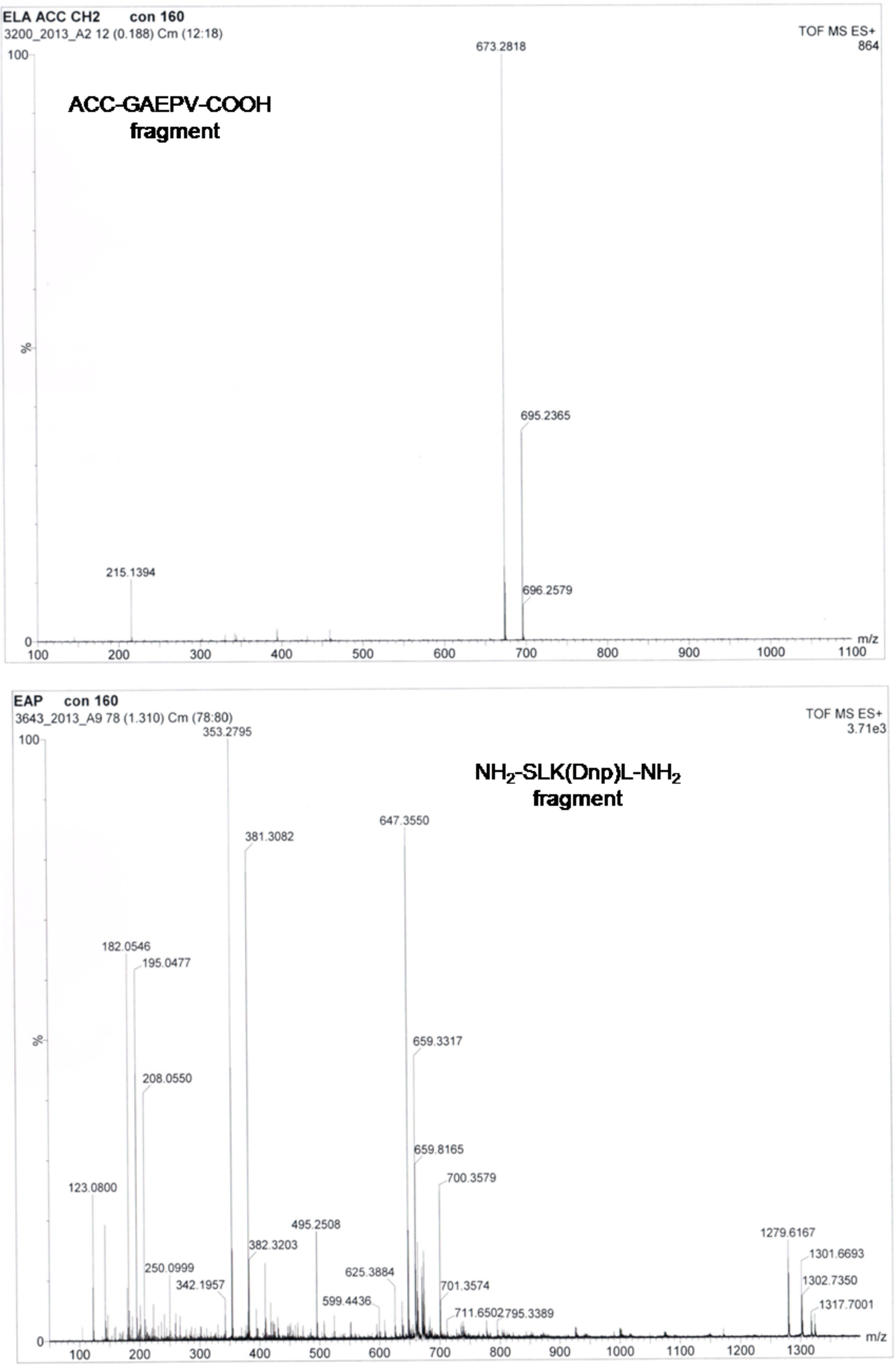

Figure S 14 The HR-MS analysis of the ACC-GAEPV/SLK(dnp)L-NH_2 cleavage fragments: N-terminal ACC-GAEPV-COOH and C-terminal NH_2-SLK(Dnp)L-NH_2 produced by human neutrophil elastase.

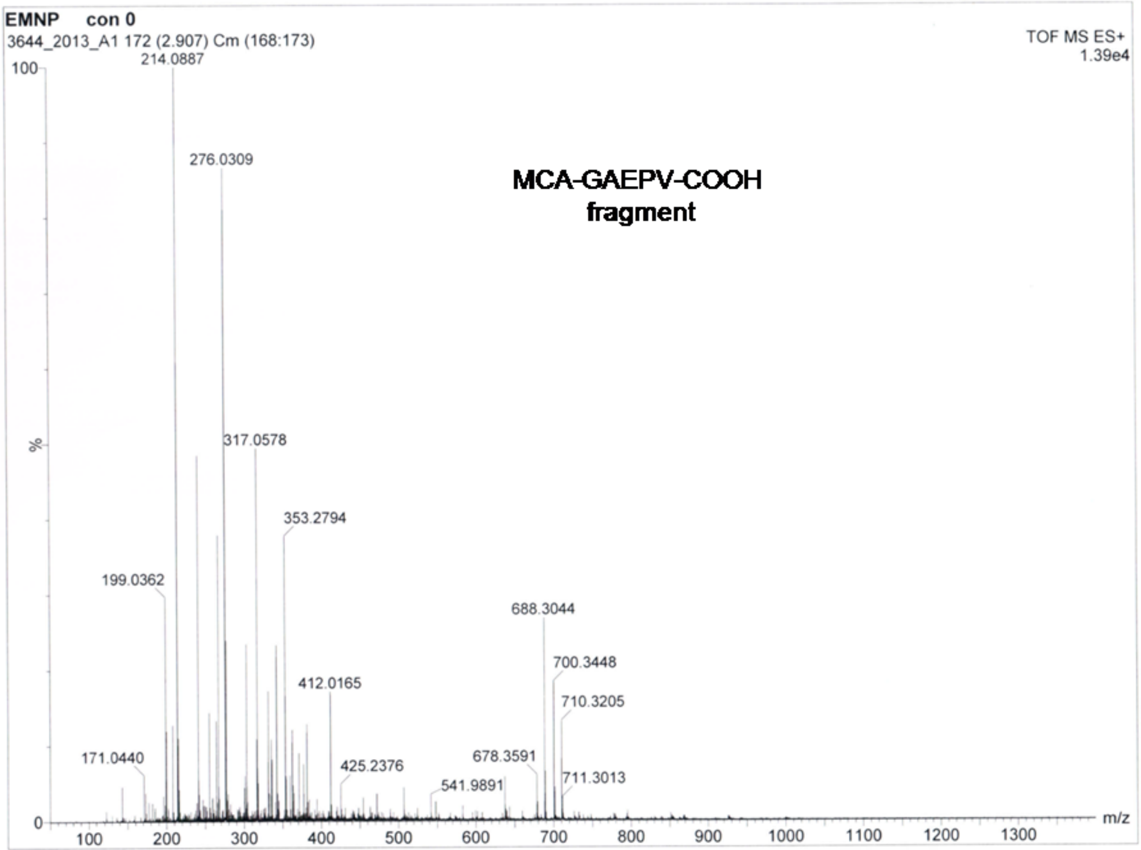

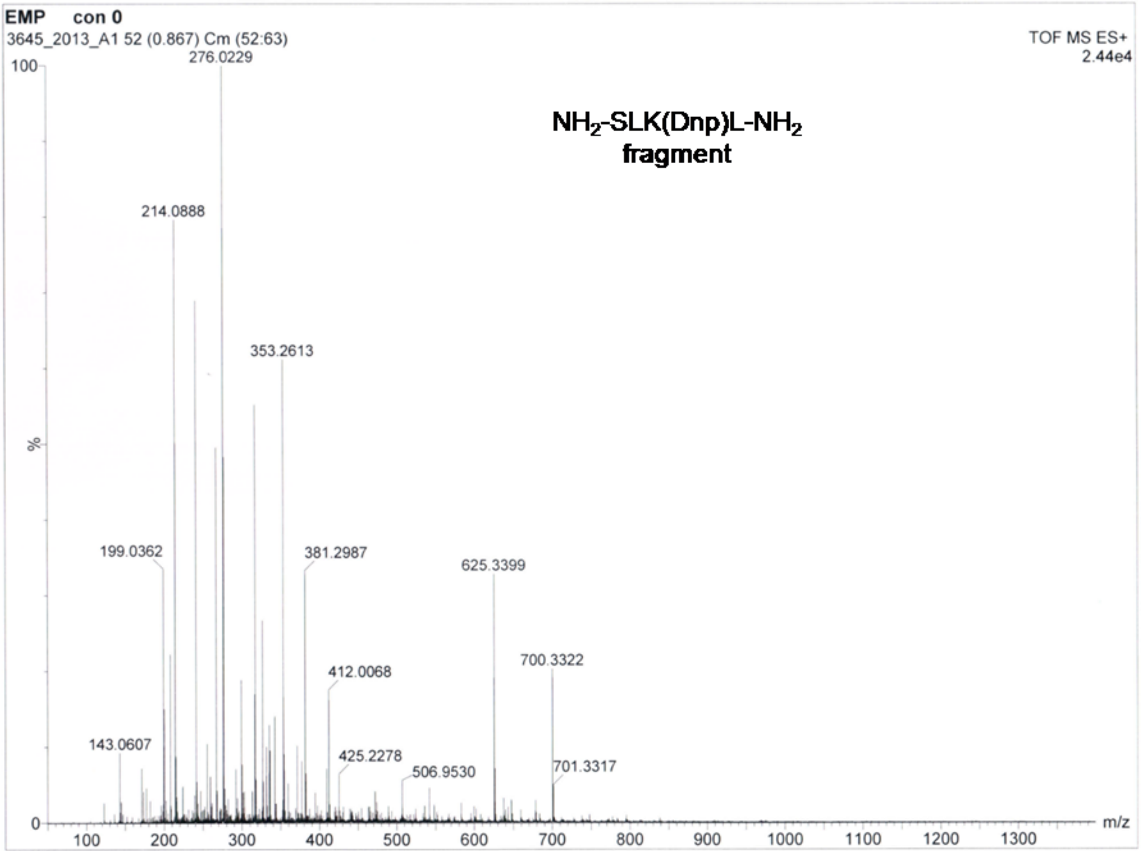

Figure S 15 The HR-MS analysis of the MCA-GAEPV/SLK(dnp)L-NH_2 cleavage fragments: N-terminal MCA-GAEPV-COOH and C-terminal NH_2-SLK(Dnp)L-NH_2 produced by human neutrophil elastase.

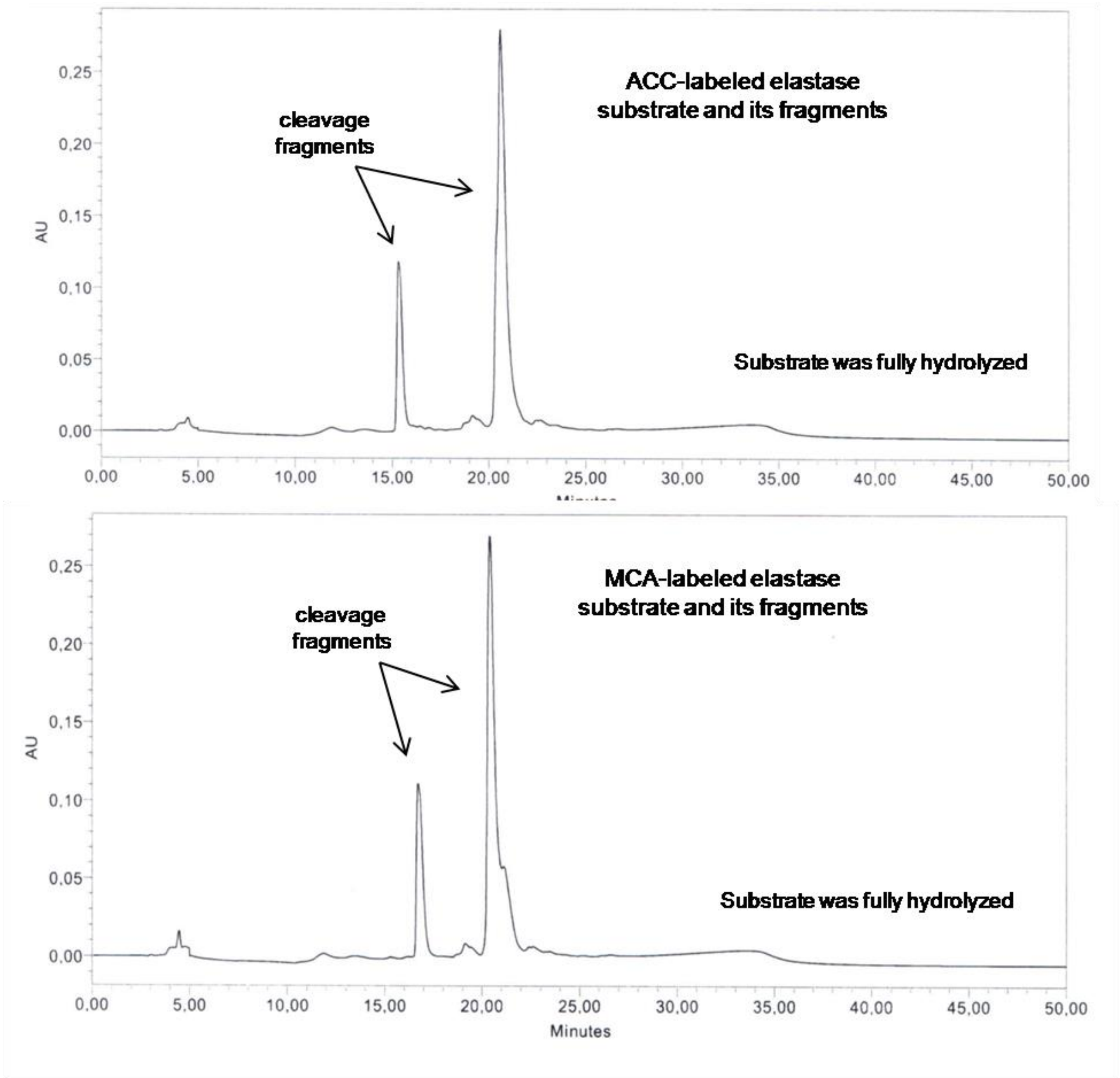

Figure S 16 The HPLC analysis of ACC- and MCA-labeled internally quenched substrates (ACC/MCA-GAEPVSLK(dnp)L-NH_2) for elastase. The cleavage fragments indicated in the figure were separated by semi-preparative HPLC and subjected to HR-MS analysis to confirm the elastase cleavage site.

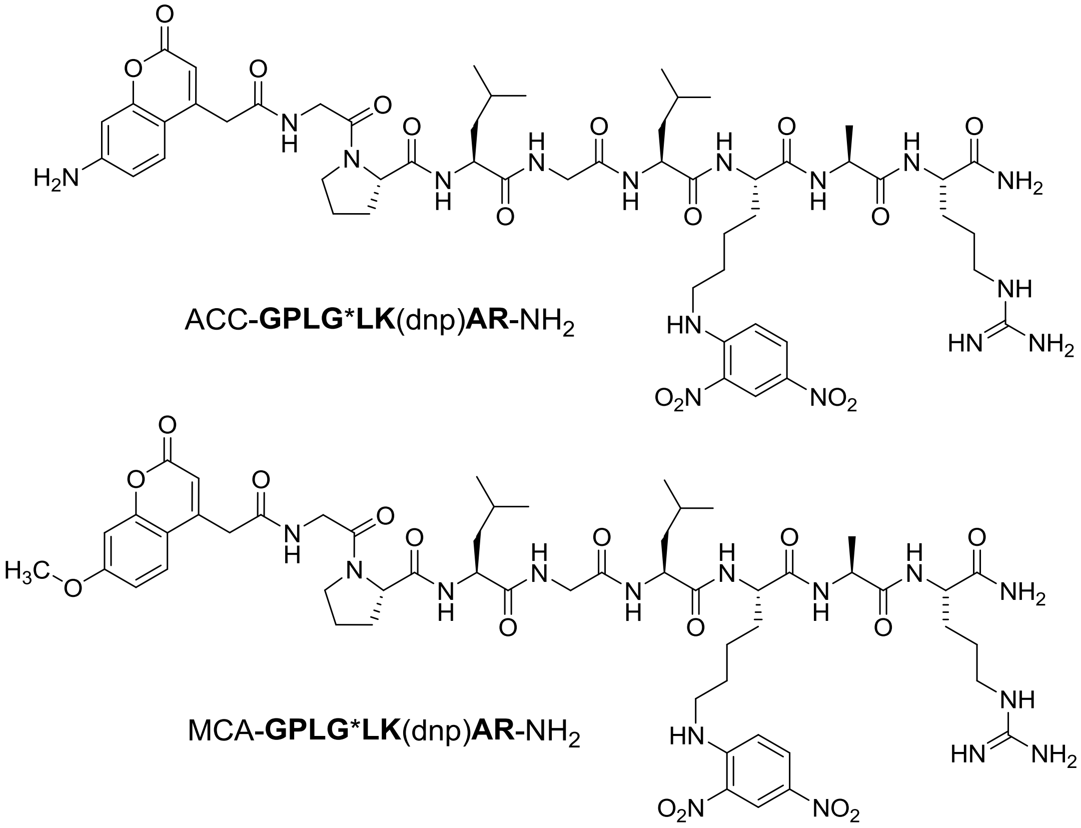

Figure S 17 The structures of two internally quenched (ACC and MCA-labeled) substrates for the investigation of MMP-2 and MMP-9.

substrate / peptide fragment	[M + H$^+$] expected	[M + H$^+$] found
ACC-GPLG*LK(dnp)AR-NH_2	1177.5754	1177.5743
ACC-GPLG-OH	544.2407	544.2413
NH_2-LK(dnp)AR-NH_2	652.3531	652.3559
MCA-GPLG*LK(dnp)AR-NH_2	1192.5751	1192.5773
MCA-GPLG-OH	581.2223 (+Na$^+$)	581.2192 (+Na$^+$)
NH_2-LK(dnp)AR-NH_2	652.3531	652.3550

Table S 5 The HR-MS analysis of the ACC- and MCA-labeled internally quenched substrates and their N-terminal and C-terminal cleavage fragments produced by MMP-2.

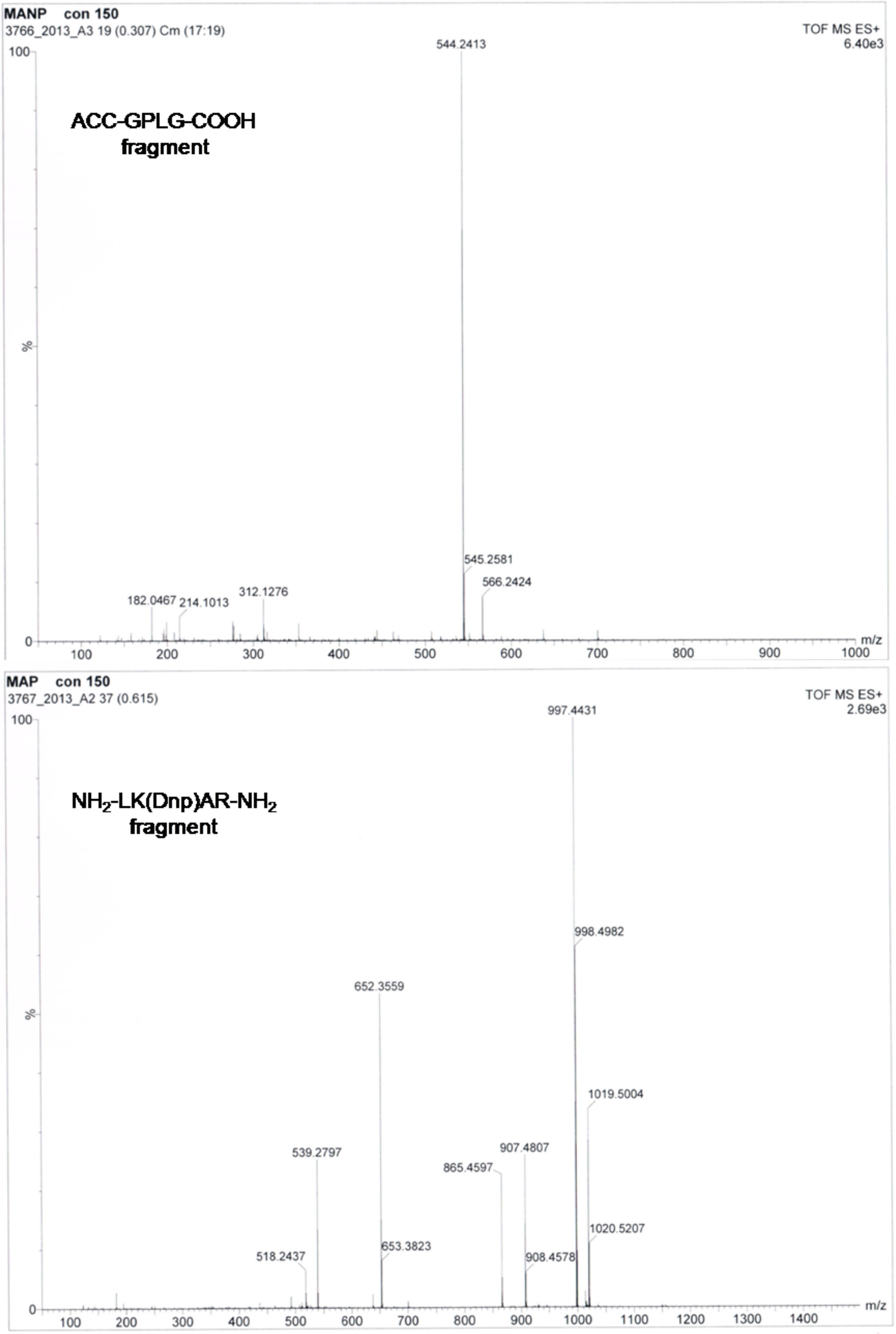

Figure S 18 The HR-MS analysis of the ACC-GPLG/LK(dnp)AR-NH_2 cleavage fragments: N-terminal ACC-GPLG-COOH and C-terminal NH_2-LK(Dnp)AR-NH_2 produced by MMP-2.

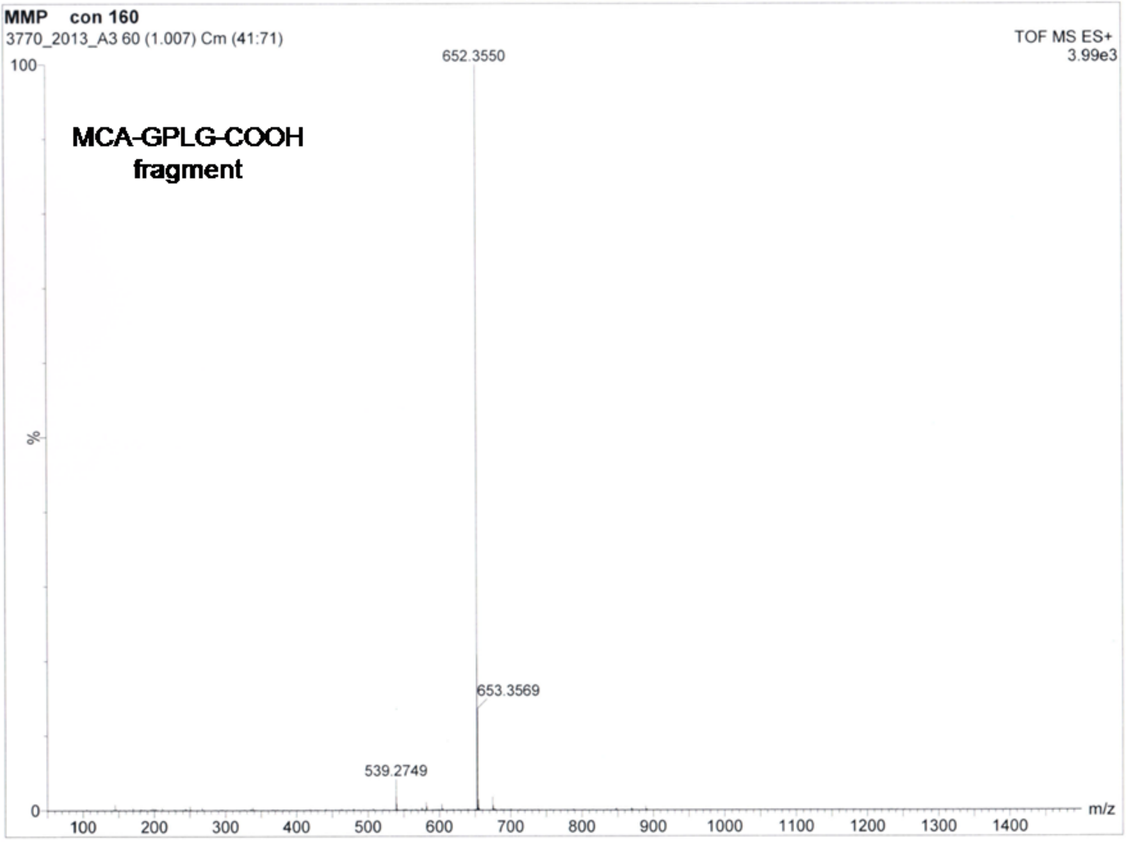

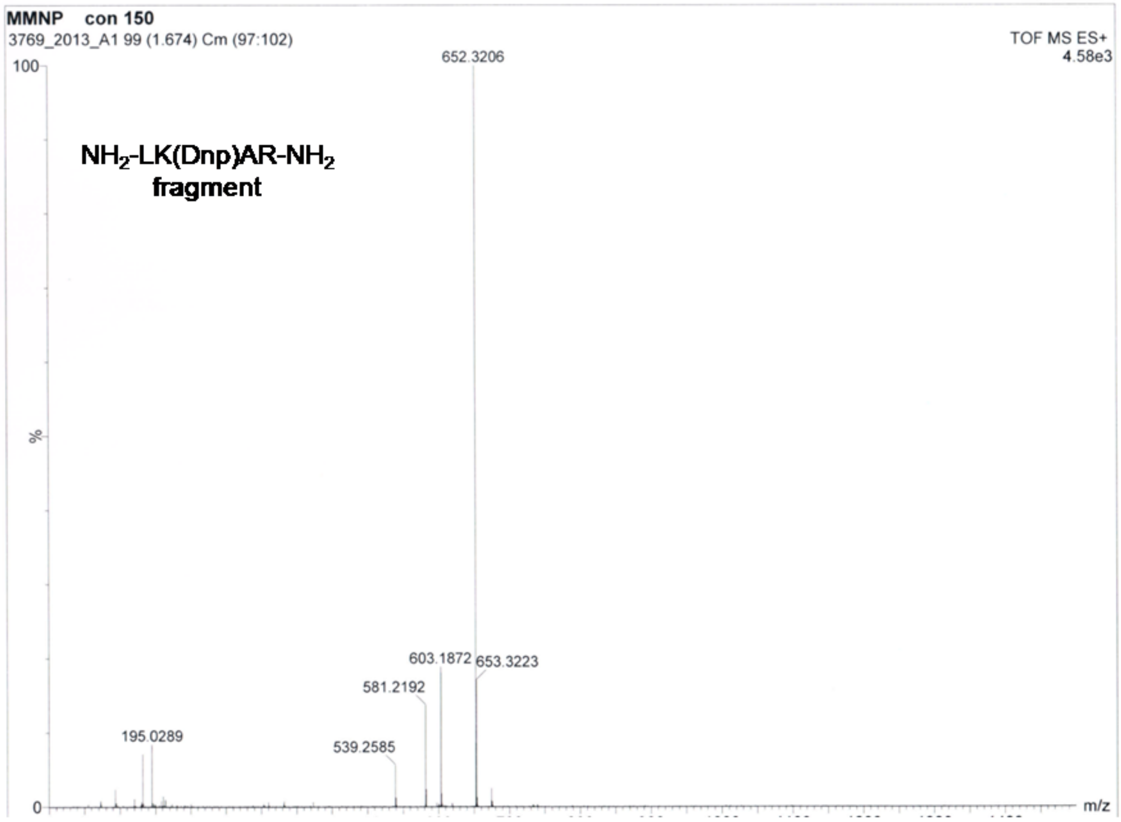

Figure S 19 The HR-MS analysis of the MCA-GPLG/LK(dnp)AR-NH_2 cleavage fragments: N-terminal MCA-GPLG-COOH and C-terminal NH_2-LK(Dnp)AR-NH_2 produced by MMP-2.

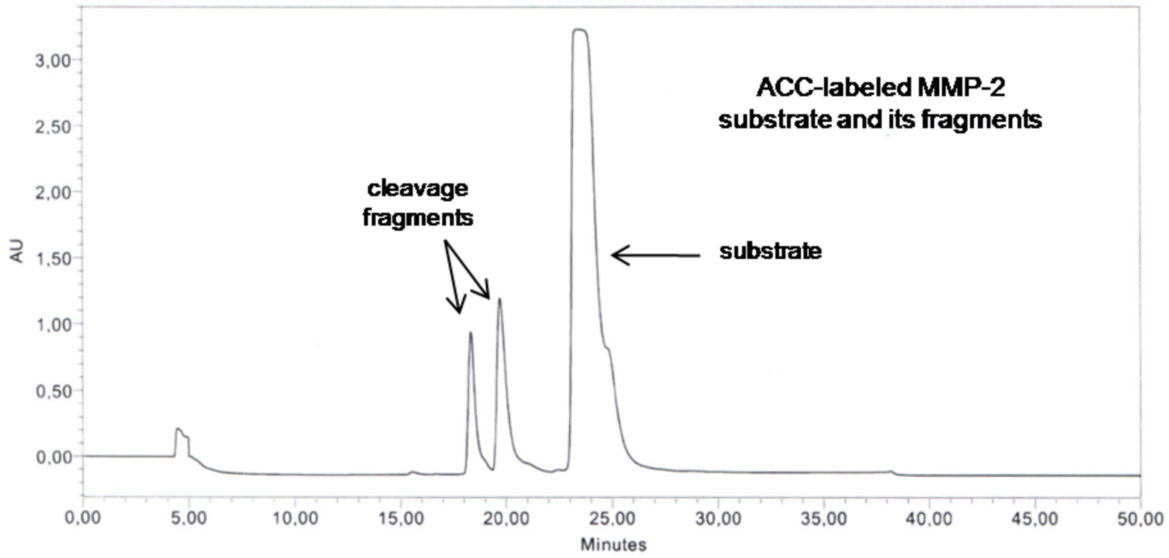

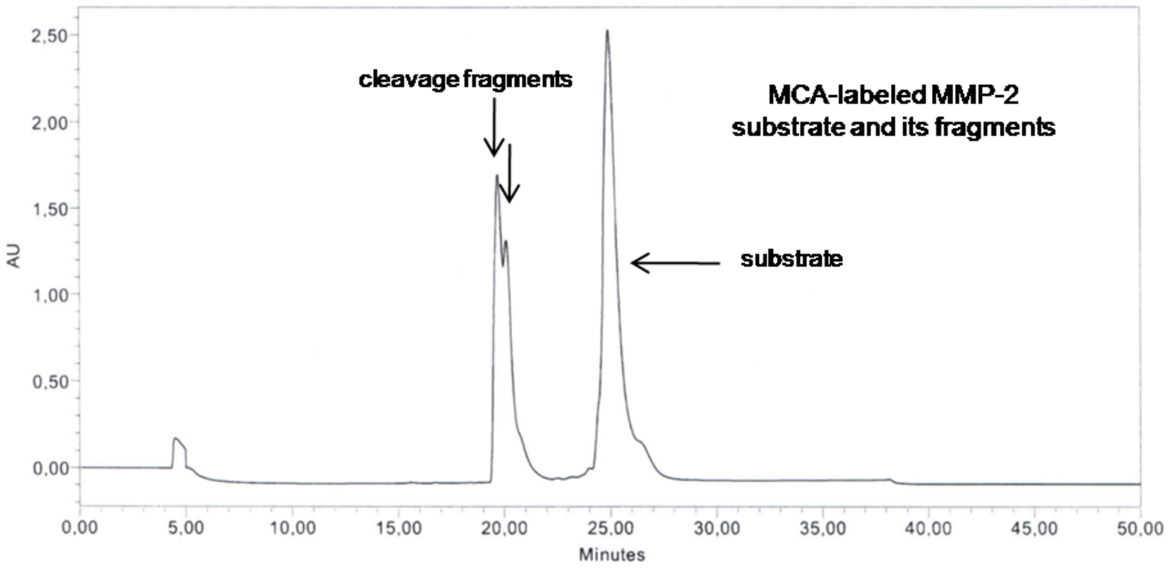

Figure S 20 The HPLC analysis of ACC- and MCA-labeled internally quenched substrates (ACC/MCA-GPLGLK(dnp)AR-NH_2) for MMP-2. The cleavage fragments indicated in the figure were separated by semi-preparative HPLC and subjected to HR-MS analysis to confirm the MMP-2 cleavage site.

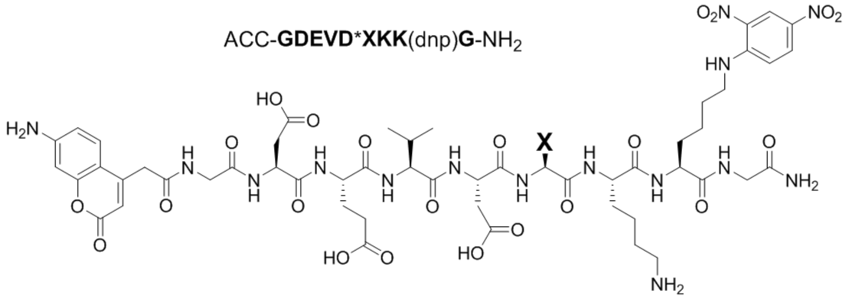

X (P1` position) =

1	Ala	11	Lys
2	Arg	12	Nle
3	Asn	13	Phe
4	Asp	14	Pro
5	Gln	15	Ser
6	Glu	16	Thr
7	Gly	17	Trp
8	His	18	Tyr
9	Ile	19	Val
10	Leu	20	Met

Figure S 21 The architecture of the internally quenched ACC-GDEVD/XKK(dnp)G-NH_2 library for the investigation of caspases`-3, -6, and -7 substrate specificity in P1` position. After the synthesis these substrates were 65-95% percent pure, so all of them were purified using semi-preparative HPLC, to obtain at least 95% purity for each individual substrate.

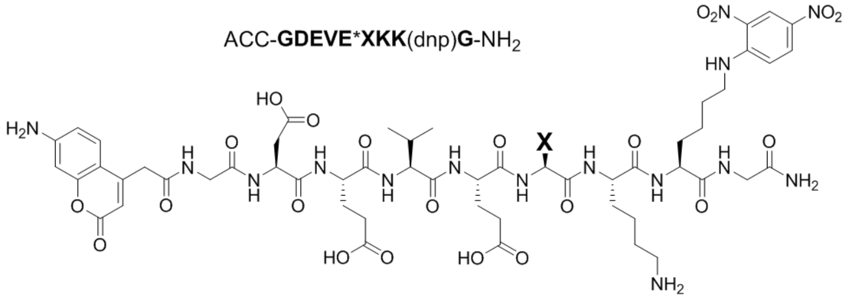

X (P1` position) =

1	Ala	11	Lys
2	Arg	12	Nle
3	Asn	13	Phe
4	Asp	14	Pro
5	Gln	15	Ser
6	Glu	16	Thr
7	Gly	17	Trp
8	His	18	Tyr
9	Ile	19	Val
10	Leu	20	Met

Figure S 22 The architecture of the internally quenched ACC-GDEVE/XKK(dnp)G-NH_2 library for the investigation of caspases`-3, -6, and -7 substrate specificity in P1` position. After the synthesis these substrates were 65-95% percent pure, so all of them were purified using semi-preparative HPLC, to obtain at least 95% purity for each individual substrate.

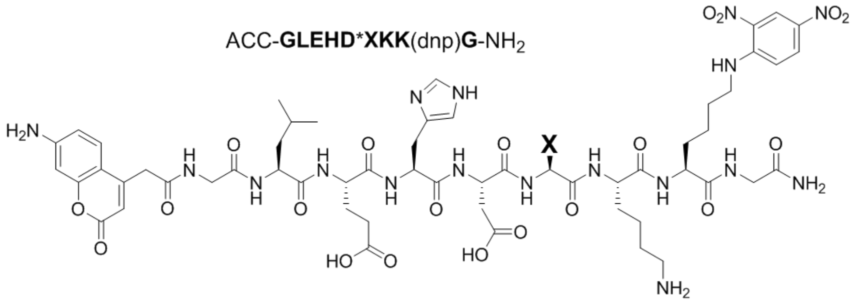

X (P1` position) =

1	Ala	11	Lys
2	Arg	12	Nle
3	Asn	13	Phe
4	Asp	14	Pro
5	Gln	15	Ser
6	Glu	16	Thr
7	Gly	17	Trp
8	His	18	Tyr
9	Ile	19	Val
10	Leu	20	Met

Figure S 23 The architecture of the internally quenched ACC-GLEHD/XKK(dnp)G-NH_2 library for the investigation of caspases`-8, -9, and -10 substrate specificity in P1` position. After the synthesis these substrates were at least 92% percent pure, so all the substrates were used for the kinetic studies without further purification.

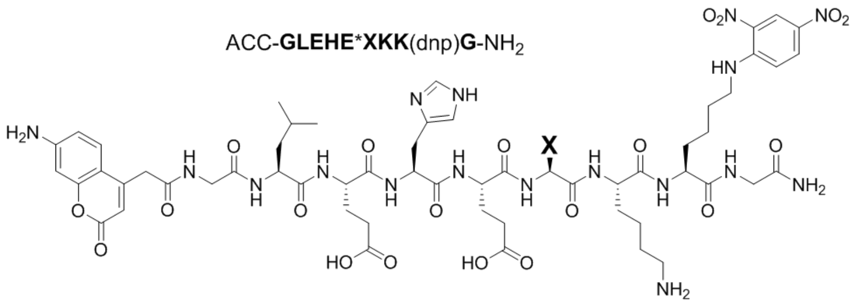

X (P1` position) =			
1	Ala	11	Lys
2	Arg	12	Nle
3	Asn	13	Phe
4	Asp	14	Pro
5	Gln	15	Ser
6	Glu	16	Thr
7	Gly	17	Trp
8	His	18	Tyr
9	Ile	19	Val
10	Leu	20	Met

Figure S 24 The architecture of the internally quenched ACC-GLEHE/XKK(dnp)G-NH_2 library for the investigation of caspases`-8, -9, and -10 substrate specificity in P1` position. After the synthesis these substrates were at least 92% percent pure, so all the substrates were used for the kinetic studies without further purification.

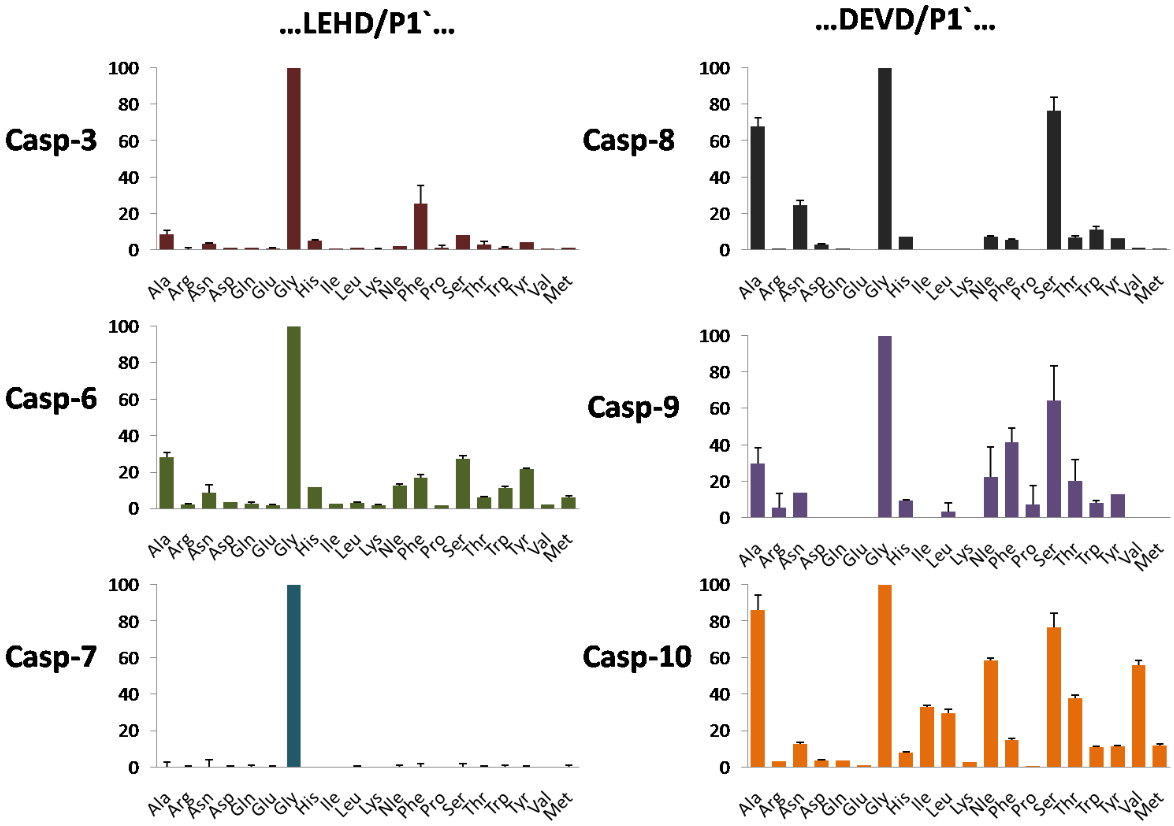

Figure S 25 The P1` substrate specificity profiles of six caspases determined with the use of P1-Asp internally quenched fluorogenic substrates libraries (ACC-G**XXX**D/P1`KK(dnp)G) containing in P4-P2 region non-optimal caspase cleavage motifs: **LEH**D for caspases-3, -6, and -7 and **DEV**D for caspases -8, -9, and -10. Library concentration 10μM. The x axis presents amino acids in three letter code, and the y axis displays the average relative activity expressed as a percentage of the best amino acid.

	k_{cat}, s^{-1}	K_M, μM	k_{cat}/K_M, $M^{-1}s^{-1}$
**Caspase-8**			
LEHD/G	0.8503	13.01	65373
LEHD/A	0.3009	17.69	17017
LEHD/S	0.4269	14.59	29261
LEHE/G	0.002985	22.68	131
LEHE/A	0.000325	19.94	16
LEHE/S	0.001045	16.58	63
**Caspase-9**			
LEHD/G	0.0755	19.79	3817
LEHD/A	0.0267	18.79	1425
LEHD/S	0.0286	17.54	1636
LEHE/G	0.0107	20.40	525
LEHE/A	0.00159	18.24	87
LEHE/S	0.00239	18.11	132
**Caspase-10**			
LEHD/G	0.1644	24.42	6742
LEHD/A	0.0990	23.26	4258
LEHD/S	0.1061	27.08	3917
LEHE/G	0.01065	24.21	441
LEHE/A	0.00135	23.44	58
LEHE/S	0.00158	30.03	52

Table S 6 The kinetic parameters of six internally quenched substrates (ACC-GLEHD(G/A/S)KK(dnp)G-NH_2 and ACC-GLEHE(G/A/S)KK(dnp)G-NH_2) measured toward caspases-8, -9, and -10.
